# Whole-genome bisulfite sequencing of cell-free DNA unveils age-dependent and ALS-associated methylation alterations

**DOI:** 10.1186/s13578-025-01366-1

**Published:** 2025-02-20

**Authors:** Yulin Jin, Karen N Conneely, Wenjing Ma, Robert K. Naviaux, Teepu Siddique, Emily G. Allen, Sandra Guingrich, Robert M. Pascuzzi, Peng Jin

**Affiliations:** 1 Department of Human Genetics, Emory University School of Medicine, Atlanta, GA 30322 USA; 2 Department of Biostatistics, University of Michigan, Ann Arbor, MI 48109 USA; 3 Departments of Medicine, Pediatrics, and Pathology, and the Mitochondrial and Metabolic Disease Center (MMDC), School of Medicine, University of California San Diego, San Diego, CA 92103 USA; 4 Feinberg School of Medicine, Northwestern University, Chicago, IL 60611 USA; 5 Department of Neurology, Indiana University School of Medicine, Indianapolis, IN 46202 USA

## Abstract

**Background:**

Cell-free DNA (cfDNA) in plasma carries epigenetic signatures specific to tissue or cell of origin. Aberrant methylation patterns in circulating cfDNA have emerged as valuable tools for noninvasive cancer detection, prenatal diagnostics, and organ transplant assessment. Such epigenetic changes also hold significant promise for the diagnosis of neurodegenerative diseases, which often progresses slowly and has a lengthy asymptomatic period. However, genome-wide cfDNA methylation changes in neurodegenerative diseases remain poorly understood.

**Results:**

We used whole-genome bisulfite sequencing (WGBS) to profile age-dependent and ALS-associated methylation signatures in cfDNA from 30 individuals, including young and middle-aged controls, as well as ALS patients with matched controls. We identified 5,223 age-related differentially methylated loci (DMLs) (FDR < 0.05), with 51.6% showing hypomethylation in older individuals. Our results significantly overlapped with age-associated CpGs identified in a large blood-based epigenome-wide association study (EWAS). Comparing ALS patients to controls, we detected 1,045 differentially methylated regions (DMRs) in gene bodies, promoters, and intergenic regions. Notably, these DMRs were linked to key ALS-associated pathways, including endocytosis and cell adhesion. Integration with spinal cord transcriptomics revealed that 31% of DMR-associated genes exhibited differential expression in ALS patients compared to controls, with over 20 genes significantly correlating with disease duration. Furthermore, comparison with published single-nucleus RNA sequencing (snRNA-Seq) data of ALS demonstrated that cfDNA methylation changes reflects cell-type-specific gene dysregulation in the brain of ALS patients, particularly in excitatory neurons and astrocytes. Deconvolution of cfDNA methylation profiles suggested altered proportions of immune and liver-derived cfDNA in ALS patients.

**Conclusions:**

cfDNA methylation is a powerful tool for assessing age-related changes and ALS-specific molecular dysregulation by revealing perturbed locus, genes, and the proportional contributions of different tissues/cells to the plasma. This technique holds promise for clinical application in biomarker discovery across a broad spectrum of neurodegenerative disorders.

**Supplementary Information:**

The online version contains supplementary material available at 10.1186/s13578-025-01366-1.

## Background

Neurodegenerative diseases (NDD) are characterized by the progressive loss of neuronal function, resulting in symptoms such as impaired movement and cognitive decline. Amyotrophic lateral sclerosis (ALS) is a rare and severe neurodegenerative disorder that primarily affects motor neurons in the brain and spinal cord. This neuronal degeneration leads to muscle weakness, paralysis, and ultimately, respiratory failure, while cognitive function is typically preserved until the later stages. Approximately 5–10% of ALS cases are familial (FALS) and are linked to genetic mutations, such as the hexanucleotide repeat expansion in the *C9ORF72* gene [1, 2]. However, the majority of cases are sporadic (SALS), with polygenic and multifactorial etiologies.

Patients who develop ALS often experience diagnostic delays. The main challenge lies in its non-specific symptoms during the initial stages, frequently overlapping with other pathological conditions. As the disease progresses, the degeneration of neurons and motor impairments become more complex, making it more difficult to distinguish between different ALS phenotypes [3]. Currently, there are no definitive biomarkers or reliable blood tests for ALS, making it difficult to detect the disease before significant neuronal damage has occurred [3]. Although cerebrospinal fluid (CSF) analysis via lumbar puncture can aid in early detection, the procedure is invasive, uncomfortable, and potentially risky. Therefore, there is a critical need for non-invasive and accurate diagnostic methods that enable earlier detection and more efficient clinical trial evaluations [4].

Cell-free DNA (cfDNA), obtainable through non-invasive blood sampling, offers a potential solution to these diagnostic challenges. cfDNA consists of short nucleic acid fragments (usually 160–180 bp) released into the bloodstream from dying cells due to normal turnover or pathological conditions. While most cfDNA molecules derive from blood cells, elevated levels of disease-associated cfDNA fragments from the affected tissue or cell types are often detectable in circulation [5]. Currently, cfDNA is increasingly recognized as a promising biomarker in various fields, including early cancer detection [6–9], fetal genetic abnormality identification [10–13], prenatal diagnostics [14–16], infectious disease screening [17–19], and organ transplantation assessment [20–22]. However, its potential in neurodegenerative diseases remains largely unexplored.

Research into nongenetic signatures of cfDNA has advanced our understanding of the biology of circulating DNA and broadened its diagnostic applications beyond genetic markers alone. In particular, DNA methylation’s tissue- and cell-type-specific nature makes cfDNA an informative tool for tracing the origins of cell death [5, 23]. During ALS pathogenesis, DNA methylation exhibits dynamic alterations and is influenced by genetic factors and environmental stimuli [24]. Many studies have reported aberrant DNA methylation patterns in CNS tissues and whole blood from ALS patients [25–31]. Despite these insights, the cfDNA methylome in ALS remains largely uncharacterized. A direct comparison of the cfDNA methylation profiles between ALS patients and healthy controls could provide valuable insights into the development of biomarkers for this disease.

DNA methylation plays an important role in regulating gene expression and genomic architecture. The rapid advancement of single-cell technologies has provided comprehensive reference maps for cell type-specific gene regulation in the CNS tissues of ALS [32, 33]. Correlating cfDNA methylation signatures with these maps could facilitate biomarker development, particularly in neurodegenerative disorders with limited access to brain tissue. Furthermore, cfDNA methylation profiling requires only a small amount of DNA, and recent technological advances have made it possible to identify methylation biomarkers using high-throughput approaches reliably. These characteristics make cfDNA methylation an attractive tool for biomarker development in ALS and other NDDs.

To address these limitations, we employed whole-genome bisulfite sequencing(WGBS) to analyze cfDNA methylation in healthy individuals across various age groups and ALS patients. We identified age-related DMLs in cfDNA and compared these DMLs to age-related CpG sites detected in a large epigenome-wide association study (EWAS) using blood samples [34]. In ALS cohorts, we characterized the cfDNA methylome and determined differentially methylated regions (DMRs) in cfDNA between ALS and control subjects. We investigated the correlation of these DMRs with gene dysregulation in ALS patient-derived CNS tissues at both the bulk and cellular levels. Additionally, we assessed whether age-associated CpGs identified from cfDNA were more likely to overlap with ALS-associated DMRs than other CpGs. Finally, we performed a deconvolution analysis to determine the contributions of various tissues and cell types to the cfDNA pools. Through this comprehensive analysis of epigenetic signatures in cfDNA, our study demonstrates that cfDNA-derived DMRs can reflect both age-related epigenetic changes and gene dysregulation signatures in ALS-affected CNS tissues, offering valuable insights for biomarker discovery across a broad spectrum of neurodegenerative disorders.

## Methods

### Study subjects

Plasma samples from 10 ALS patients and 8 healthy controls were obtained with informed consent through the Indiana University School of Medicine IRB Project# 2,009,735,565 and processed through the Mitochondrial and Metabolic Disease Center (MMDC) at the University of California, San Diego, IRB Project #140,072. Additionally, 30 healthy young and middle-aged participants were recruited with informed consent, and the study received approval at Emory University and Northwestern University, respectively. The datasets used in this study are summarized in detail in Supplementary Table 1.

### Blood processing and cfDNA extraction

For each subject, 4 ml peripheral blood was collected using EDTA-containing tubes, and the blood was centrifuged at 1600 g, 4 °C for 15 min, then the plasma portion was harvested and re-recentrifuged at 13,000 g, 4 °C for 15 min to remove blood cells. Plasma samples were stored at -80 °C until further usage. Circulating cfDNA was extracted from 600ul of plasma using the QIAamp MinElute ccfDNA Midi Kit (Qiagen #55284). Briefly, 35 µL proteinase K solution and 18 µL Magnetic Bead Suspension were added to the plasma. Nest, 90ul Bead Binding Buffer was added to the mixture and incubated at room temperature for 15 min with gentle shaking end-over-end. Following magnetic separation, the supernatant was discarded, and the magnetic beads were incubated with 200 µL of Bead Elution Buffer for 5 min on a thermomixer at room temperature and 300 rpm, then placed on the magnetic separation rack. The supernatant was transferred to a new Bead Elution Tube, mixed with 300 µL of Buffer ACB, and applied to a QIAamp UCP MinElute Column, followed by centrifugation at 6000 x g for 1 min. The column was then washed with 500 µL of ACW2 buffer and incubated at 56 °C for 3 min with the lid open. Finally, cfDNA was eluted with 15 µL of Nuclease-Free Water (Ambion #AM9938) and quantified using the Qubit dsDNA HS Assay Kit (Invitrogen #Q32851) on a Qubit 3 Fluorometer.

### WGBS library preparation and sequencing

The EZ DNA Methylation Gold kit (Zymo Research #D5005) was used for sodium bisulfite conversion prior to library preparation. The Accel-NGS Methyl-Seq (Swift BioSciences #30024) kit was used for library preparation according to the manufacturer’s protocols. Post-library QC was performed with the Qubit dsDNA High Sensitivity fluorometric assay (Invitrogen #Q32854) and BioAnalyzer DNA 1000 chips (Agilent #5067 − 1504). Paired end sequencing (2 × 150) was performed on an Illumina NovaSeq 6000 System (Illumina). A PhiX library was spiked in at 25% for each sequencing library.

### WGBS data processing

All our WGBS data were processed according to the ENCODE consortium guidelines (Nature 2012, ENCODE Project). Quality of the fastq files was assessed using FastQC (v0.11.9). Adaptors were trimmed from the paired end fastq files using Trimmomatic (v0.39) [35]. Ten basepairs were trimmed from the beginning of both R1 and R2 using Cutadapt v2.10 to eliminate any artifictual cytosine methylation introduced because of filling in overhangs during end repair steps and low-quality bases due to bisulfite treatment. The processed reads were aligned to the human genome (hg19), deduplicated, examined for coverage, and extracted to CG count matrix using Bismark v0.19.0 [36]. Briefly, reference sequences were converted to map both cytosine and thymine. The mapping of WGBS libraries was performed with the bismark module followed by duplication removal using deduplicate_bismark. Methylation calling was processed using a module called Methylation Extractor. The modules of bismark2bedGraph and coverage2cytosine were used to extract CpG sites and estimate their methylation levels. Genome-wide methylation levels were estimated as the weighted methylation level of all mCG.

### DMR calling and annotation

DMLs and DMRs were called by utilizing the package DSS [37]. First, the DMLtest function was used to perform tests for differential methylation between groups for all CpG sites with smoothing, and DMLs were identified using the callDML function. The difference in mean methylation level between the ALS and control was computed and significance of this difference was assessed using a Wald test at each CpG site that incorporates a shrunken dispersion estimate that borrows information across the genome. DMLs were identified as those CpG sites with FDR < 0.05. Finally, the callDMR function was used to call DMRs at P-value less than 0.05, with the following criteria: each DMR has minimum length of 50 bp and included at least 4 DMLs. Next, DMRs were assigned to a given genomic feature using package Annotatr [38] in R. The enrichment of DMRs was calculated by the fold change between the percentage of DMRs located in different genetic features and the expected genomic length percentages. The expected values were determined by the genomic space occupied by the genomic features associated with DMRs by chance. For intergenic features, the associated areas were summed to obtain the length of genomic space. For other genomic features, the length of each feature was summed based on the obtained annotation information.

We used the getMeth function from the package BSseq v1.28.0 in R to calculate the average methylation levels for these obtained DMRs across all the samples. Heatmaps were generated using the pheatmap function from the pheatmap v1.0.10 package in R, with columns representing samples and rows representing DMRs, to illustrate the methylation level changes across different sample groups. Gene ontology (GO) analyses for genes associated with identified DMRs were performed using the DAVID (The Database for Annotation, Visualization and Integrated Discovery) tool [39].

### Plasma DNA methylation deconvolution

Deconvolution analysis of plasma DNA tissue mapping was performed by using cfDNAmethy (quadratic programming (QP) algorithm) as described in Feng et al. [40](https://github.com/haoharryfeng/cfDNAmethy). Briefly, the reference-panel of tissue-specific methylation markers from 14 different tissues were obtained from a previous study [41], including liver, lungs, esophagus, heart, pancreas, colon, small intestines, adipose tissues, adrenal glands, brain, and T cells, B cells, neutrophils, and placenta. Type I markers (*n* = 1013) refer to any genomic loci with methylation densities that are significantly different in one tissue compared with the mean level of other tissue types, while Type II markers (*n* = 4,807) are genomic loci that demonstrate highly variable methylation densities across all tissue types [41]. QP was applied on the methylation data of ALS cohort and reference panel to estimate tissue proportions for each subject.

### Metagene analysis

DNA methylation profiles around gene bodies were calculated and plotted by deepTools2 [42]. First, we used WiggleTools [43] to average the DNA methylation percentages of two replicates for each neuronal type. Then, the computeMatrix module from deepTools2 with scale-regions parameter was used to calculate the average scores for a specific input gene set between upstream (-10Kb) TSS (Transcription Start Site) and TES (Transcription End Site) downstream (+ 10Kb) regions. Next, based on the calculated scores, the plotProfile module was used to plot out the DNA methylation profiles around the given gene bodies.

## Results

### Age-dependent methylation signature in cfDNA

**Fig. 1:**
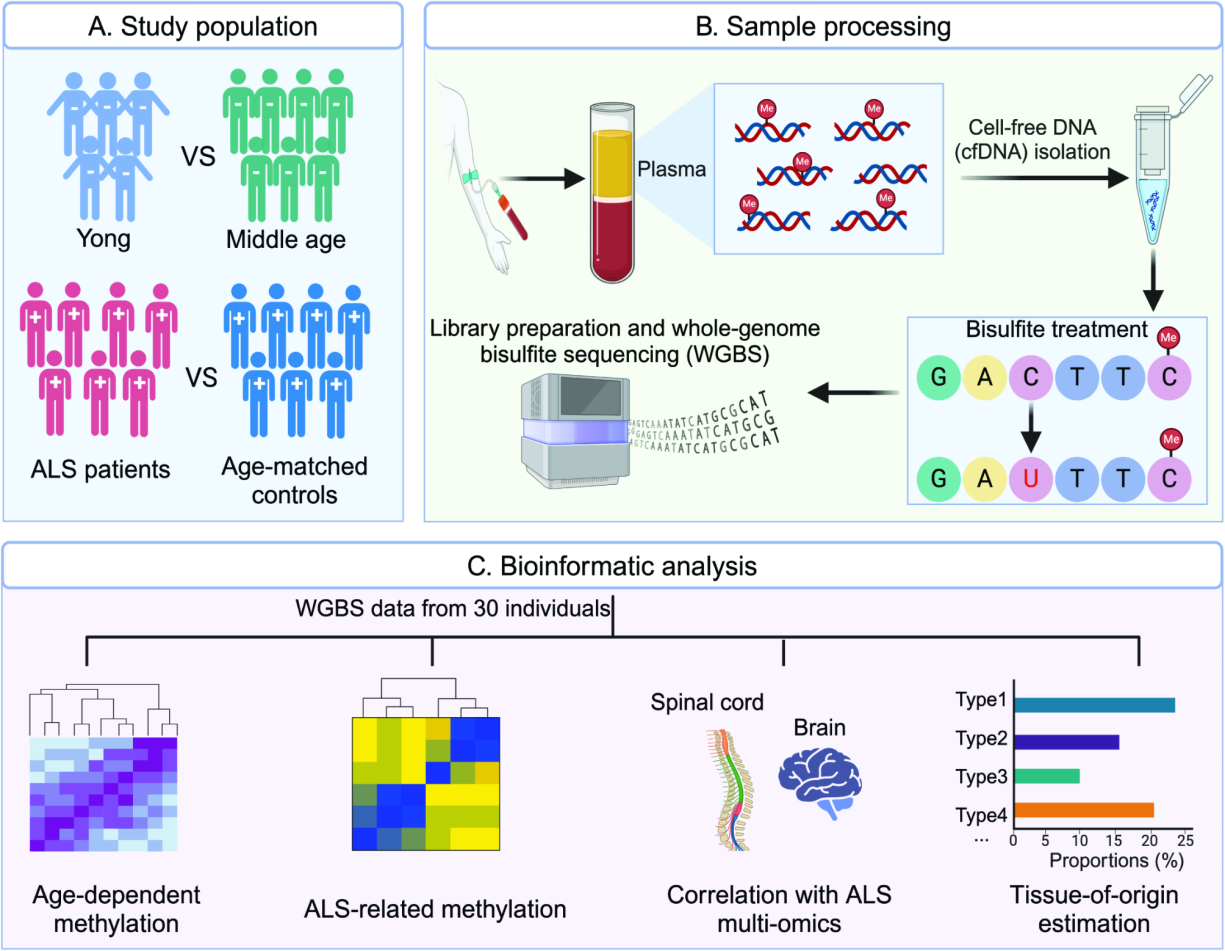
**Overview of study workflow utilizing genome-wide methylation analysis of plasma-derived cfDNA to identify age-related and ALS-associated methylation signatures.** Plasma samples were collected from 30 individuals, including healthy participants from various age groups, ALS patients, and age- and gender-matched control subjects. Methylome profiling of cfDNA was conducted using WGBS on bisulfite-treated cfDNA. Age-related and ALS-associated methylation signatures were identified through comprehensive bioinformatic analysis. Additionally, the correlation between ALS-related methylation profiles and gene dysregulation in CNS tissues derived from ALS patients was investigated at both the bulk and single-cell levels. A reference-based deconvolution analysis was performed to determine the tissue and cell-type origins of the cfDNA

**Fig. 2:**
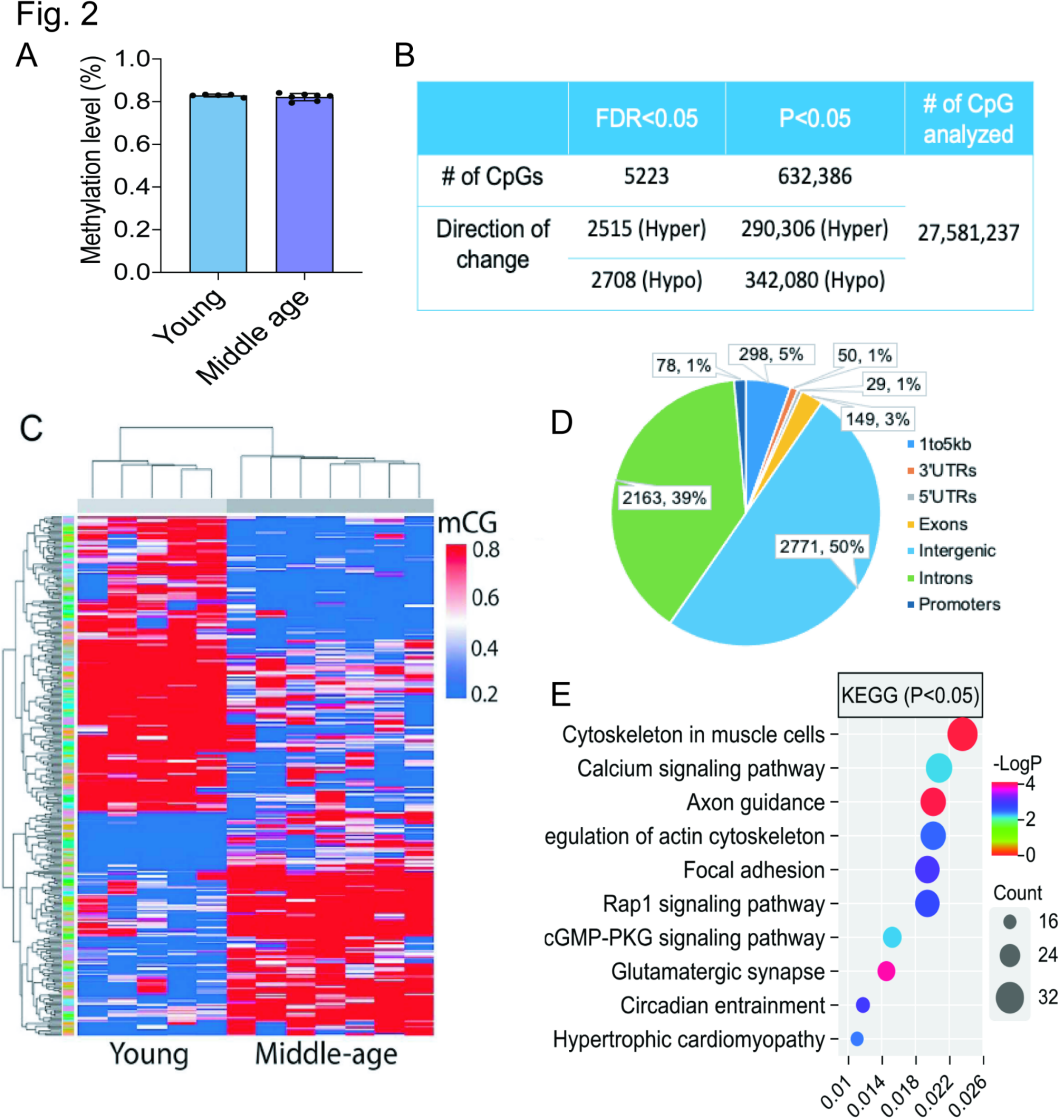
**Identification of age-related methylation features in cfDNA. (A)** Bar graph showing average genome-wide mCG levels in young and middle-aged groups. No significant difference of methylation levels was observed between groups. **(B)** Identification of DML between the two groups. **(C)** Heatmap visualization of the methylation profiles of DMLs identified between the two groups. **(D)** Genomic annotation of the identified DMLs to their percentage of each genomic feature. **(E)** KEGG analysis for DML-associated genes to examine their biological significance

A total of 30 individuals were included in our study (Fig. 1; Supplementary Table 1), and WGBS provided an average of 24.1 million CpG sites per sample. Age-related DNA methylation changes are a normal part of the aging process, such changes could also influence the onset and progression of neurodegenerative diseases like ALS. To investigate age-related differential methylation in cfDNA, we analyzed cfDNA samples from 12 control subjects, comparing five young adults (16–23 years) and seven middle-aged adults (36–63 years). The estimation of genome-wide CpG methylation levels revealed an average of 82.9% in the young group and 82.2% for aged individuals (Fig. 2A). A total of 27.5 million CpG sites were assessed, revealing 5,223 differentially methylated loci (DML) between the two age groups at FDR < 0.05 (Fig. 2B-C; Supplementary Table 2). Among these, 2,708 CpGs (51.6%) showed decreased methylation in older individuals. Most DMLs were located in intergenic and intronic regions, with ~ 8% found in regulatory regions, while approximately 8% were found in regulatory regions (Fig. 2D). Annotation and Kyoto Encyclopedia of Genes and Genomes (KEGG) pathway analysis revealed that these age-related CpGs in cfDNA were linked to genes involved in cytoskeletal organization in muscle cells, axon guidance, and the calcium signaling pathways (Fig. 2E). Additionally, 632,386 CpGs were identified as differentially methylated at *P* < 0.05 (Fig. 2B).

We then compared our DMLs to age-related CpG sites identified in a large EWAS covering over 18,000 blood samples [34]. Of the 751,318 loci present in both datasets, we found significant overlap (*n* = 3,947 CpGs, OR = 2.39, *P* < 2.2 × 10⁻¹⁶) between CpGs with *P* < 0.05 in our study and CpGs with Bonferroni significance (*P* < 3.6 × 10^− 8^) in the EWAS dataset (Table 1). Moreover, CpGs with *P* < 0.05 in our study demonstrated greater-than-expected directional concordance with the EWAS findings (OR = 1.75, *P* < 2.2 × 10^− 16^, Table 1). However, among those 5,223 DML with FDR < 0.05 identified in our study, only 7 loci were present in the EWAS blood dataset, with no significant enrichment observed at these sites due to limited statistical power.

**Table 1:** Concordance in direction of shared CpG sites between cfDNA and the blood samples

	# of shared CpG	# Significant in ^citation^	Enrichment^*^	Direction in cfDNA	Direction in citation		Enrichment^**^
					(-)	(+)	
**FDR < 0.05**	7	1	OR = 1.09 *P* = 1	**(-)**	1	1	OR = 0.71, *P* = 1
				**(+)**	3	2	
***P*** **< 0.05**	14,986	3947	OR = 2.39 *P* < 2.2 × 10^− 16^	**(-)**	4299	3020	OR = 1.75, *P* < 2.2e-16
				**(+)**	3441	4226	

(-): Sites showing significantly decreased methylation in older individuals

(+): Sites showing significantly increased methylation in older individuals

* Fisher’s exact test of whether there is greater-than-expected overlap between our differentially methylated sites and those identified in [34]

** Fisher’s exact test of whether our differentially methylated sites show greater-than-expected directional concordance with those identified in [34]

### Characterization of cfDNA-derived DNA methylation landscapes in ALS

**Fig. 3:**
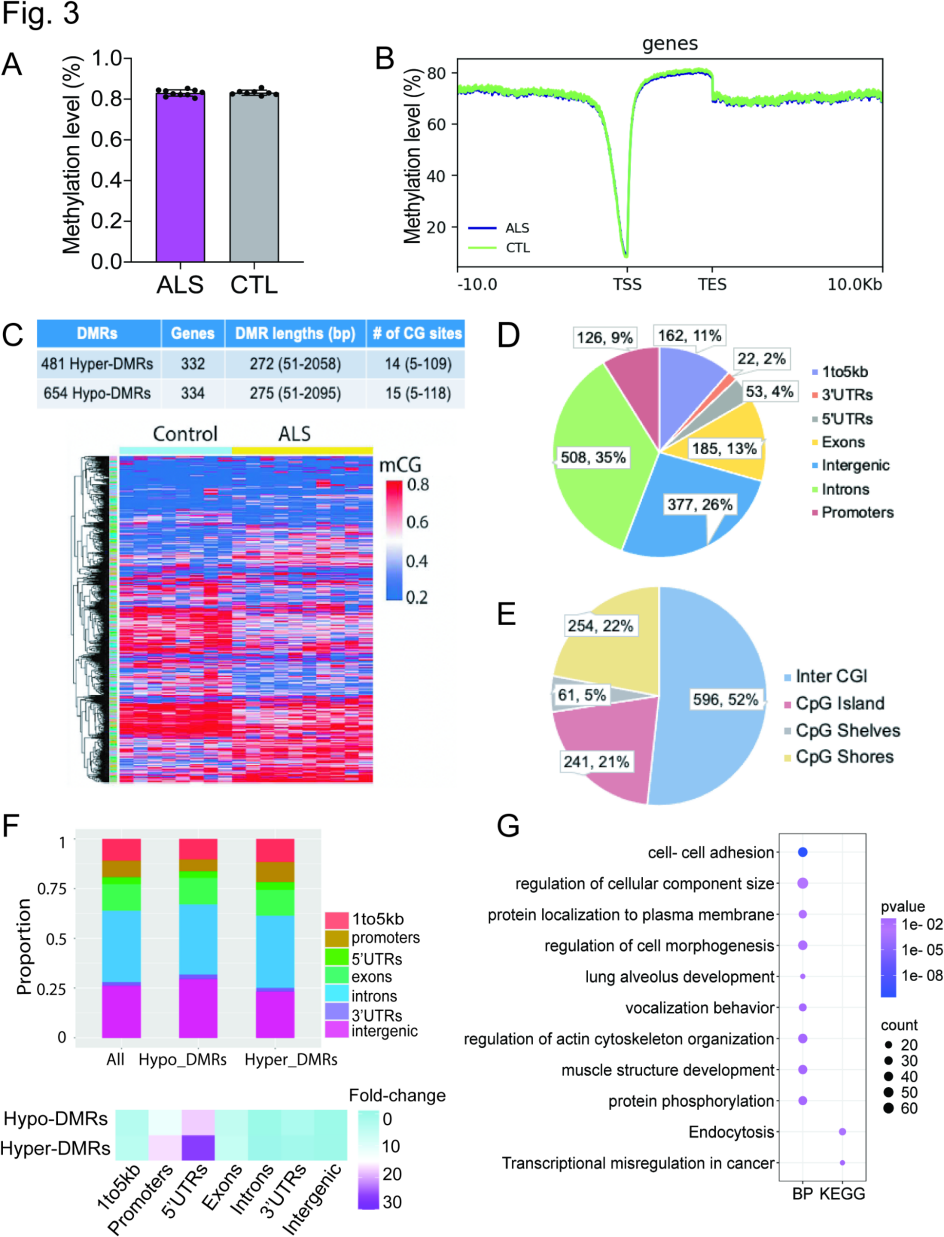
**Characterization of cfDNA-derived DNA methylation landscapes in ALS. (A)** Bar graph showing average genome-wide mCG levels in ALS and control groups. No significant difference of methylation levels was observed between groups. **(B)** Metagene plots showing mCG (top) levels across transcriptional start sites (TSS), transcriptional end sites (TES) and RefSeq gene bodies. Ten kb upstream and downstream of given genomic features were plotted. **(C)** Identification of DMRs between ALS and control groups (top), with a heatmap (bottom) visualizing the methylation profiles of the identified DMRs. **(D)** Genomic annotation of the identified DMRs on their percentage of each genomic feature. **(E)** Proportion of ALS-related DMRs within CpG islands (CGIs), CpG shelves, CpG shores, and other genomic regions. **(F)** Top: Proportion of hypermethylated DMRs and hypomethylated DMRs on their percentage of each genomic feature. Bottom: Fold enrichment of DMRs over genomic background. **(G)** GO and KEGG pathway analysis of DMR-associated genes to explore their functional relevance to ALS

In parallel, we examined cfDNA methylation profiles in a cohort of 10 ALS patients and 8 healthy individuals. All the participants are male, with an average age of 60 in both groups (Supplementary Fig. 1A; Supplementary Table 1). Global CpG methylation levels were similar between ALS patients and control individuals, averaging 83% across all samples (Fig. 3A). Metagene analysis revealed that cfDNA fragments from WGBS were enriched in coding and intergenic regions but were significantly depleted around transcription start sites (TSS) (Fig. 3B). We then examined local genomic regions exhibiting differential CpG methylation levels in cfDNA. A total of 1,045 DMRs were identified between ALS patients and controls, ranging from 51 to 2,955 base pairs in size (P-value < 0.05, methylation difference > 0.1) (Fig. 3C; Supplementary Table 3). A slightly higher number of DMRs were hypomethylated (564) compared to hypermethylated (481), with DMRs consistently distributed across all chromosomes (Supplementary Fig. 1B). The full landscape of these DMRs is illustrated by a heatmap (Fig. 3C), and as expected, the methylation signatures of these DMRs clearly distinguished ALS patients from control subjects.

Genomic annotation revealed that ALS-related DMRs were located within gene bodies, promoters, and distal intergenic areas (Fig. 3D; Supplementary Table 4), with approximately 21% of DMRs are in CpG islands (Fig. 3E). We found a higher proportion of hypermethylated DMRs in promoter regions compared to hypomethylated DMRs. In contrast, the opposite trend was observed in intergenic regions. Moreover, among the annotated genomic features, DMRs located in the 5’ UTR and promoter regions exhibited the highest level of enrichment compared to the expected value, particularly pronounced among hypermethylated DMRs (Fig. 3F), highlighting their potential regulatory roles.

To explore the functional implications of the observed methylation changes in cfDNA of ALS, we conducted GO and KEGG enrichment analyses on genes associated with DMRs. As shown in Fig. 3G, methylation changes in cfDNA were significantly associated with genes involved in the cell-cell adhesion process and endocytosis pathway (P-value < 0.01). Notably, DMRs in promoter regions were particularly enriched in the endocytosis pathway (Supplementary Fig. 1C). Since ALS affects multiple organ systems beyond the nervous system, including muscle function, digestion, and respiration, we also observed methylation changes in genes related to muscle structure development, lung alveolus development, and vocalization behavior. These findings indicate that cfDNA methylation profiles may provide valuable insights into the pathology of ALS. We also investigated whether age-associated CpGs were more likely to overlap with ALS-associated DMRs compared to other CpGs. Fisher’s exact test revealed a strong enrichment for ALS DMRs (OR = 5.8, *P* = 1.094 × 10^− 07^) (Supplementary Table 5). Specifically, 15 age-associated CpGs were found to overlap with ALS DMRs (), potentially representing critical loci where age-related epigenetic modifications intersect with ALS pathophysiology.

### Correlation of cfDNA methylation with spinal cord gene expression in ALS

**Fig. 4:**
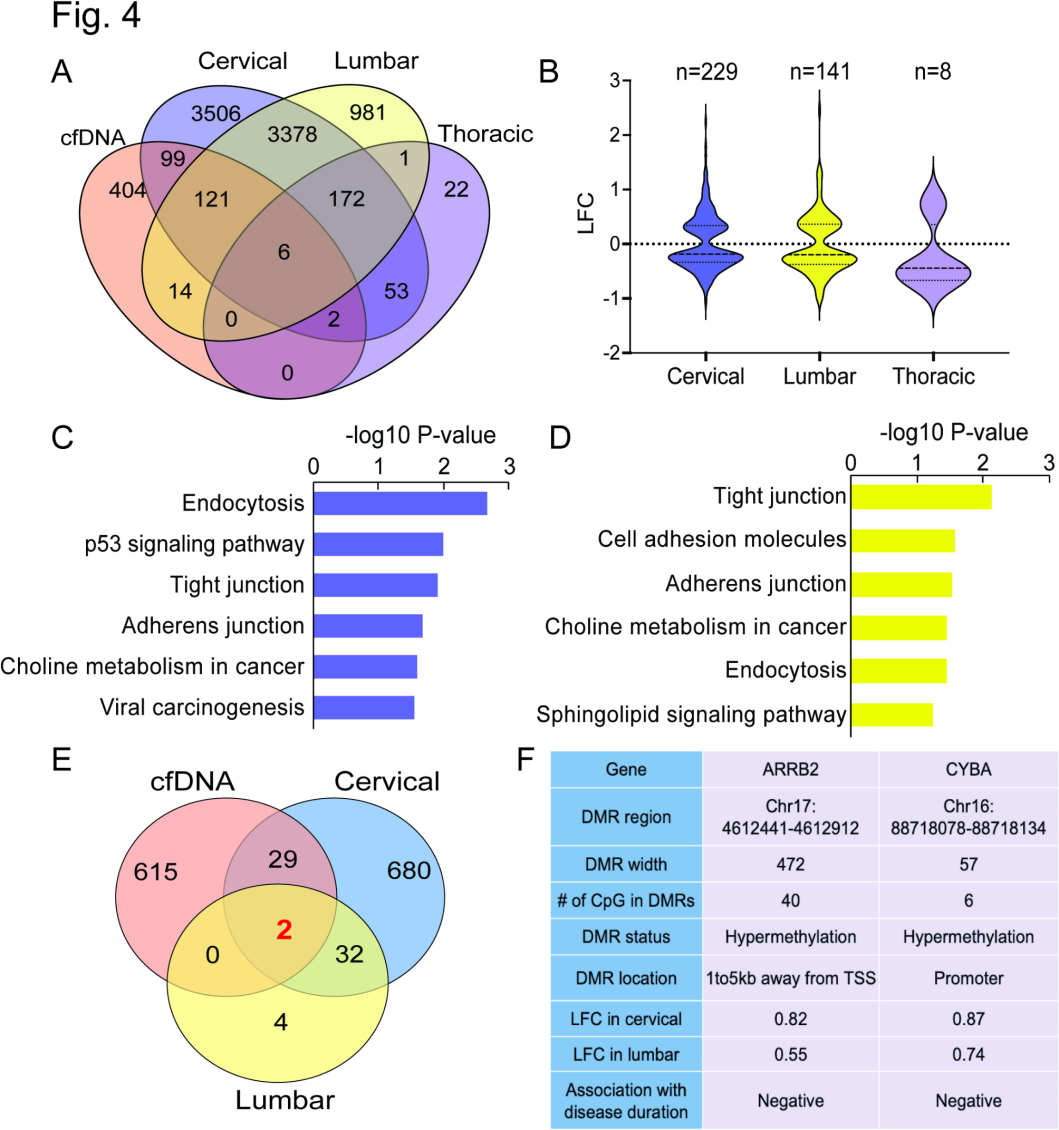
**Integration of cfDNA methylation with spinal cord gene expression in ALS** [49]. **(A)** Venn diagram illustrating the overlap between cfDNA-derived DMR-associated genes and DEGs identified in three spinal cord segments of ALS patients. **(B)** Violin plots displaying the log2 fold-change (LFC) of DEGs with differential methylation in cfDNA from the spinal cords of ALS patients compared to controls. **C-D.** KEGG pathway analysis of overlapping genes in the cervical and lumbar spinal cord segments. **E.** Venn diagram displaying the overlap between DMR-associated genes and genes linked to disease duration in ALS. **F.** Detailed information on DMRs annotated to ARRB2 and CYBA, along with their gene expression statistics in the spinal cord

DNA methylation is known to regulate gene expression in a tissue- and cell-specific manner in CNS [44–47]. The methylation patterns observed in cfDNA likely offer valuable insights into the epigenetic regulation of genes across various tissues [5, 48]. To explore this further, we integrated ALS-related DMRs with bulk gene expression data [49] from the spinal cord, a key site of ALS neurodegeneration, particularly affecting the motor neurons that control voluntary muscle movement [50]. The site of ALS onset is an important factor that can influence the distribution of molecular changes across different spinal cord segments. ALS typically begins in one region, such as the cervical, thoracic, or lumbar spinal cord, and the disease progression follows a rostrocaudal gradient, affecting different spinal cord regions over time [50]. Consequently, variation in onset location may lead to differential distributions of DEGs across spinal cord segments [49]. Our analysis revealed that 31–33% of genes linked to these DMRs showed differential expression across three spinal cord segments (cervical, lumbar, and thoracic) in ALS patients compared to controls (Fig. 4A; Supplementary Tables 6–8). Specifically, we identified 229, 141, and 8 differentially expressed genes (DEGs) in the cervical, lumbar, and thoracic regions, respectively. These genes account for approximately 3% of the DEGs reported in the referenced study. Violin plots of the log2 fold-change (LFC) for these genes demonstrated a trend toward downregulation, with median expression levels lower in the spinal cords of ALS relative to controls (Fig. 4B). KEGG pathway analysis of these overlapping genes highlighted their involvement in the endocytosis, tight junction, and adherens junction pathways (Fig. 4C-D).

In the same study, the expression of 745 and 39 genes was significantly associated with ALS disease duration (FDR < 0.05) in the cervical and lumbar spinal cords, respectively [49]. By overlapping these gene sets with our list of DMR-linked genes, we identified 31 differentially methylated genes correlated with ALS progression (Fig. 4E; Supplementary Tables 9–10). This overlap suggests that epigenetic modifications in these genes may contribute to ALS progression. Among these, ARRB2 and CYBA were consistently associated with disease duration in both spinal cord regions (Fig. 4F). ARRB2 encodes β-arrestin 2, which regulates various signaling pathways, including those involved in cell survival and apoptosis processes that are important in the pathophysiology of neurodegenerative diseases, such as Alzheimer’s disease (AD) [51, 52], frontotemporal dementia (FTD) [53], and Parkinson’s disease (PD) [54]. CYBA, which encodes the p22phox subunit of the NADPH oxidase complex, is critical for generating reactive oxygen species (ROS) and has been implicated in oxidative stress, a known contributor to ALS pathology [55]. As shown in Fig. 4F, the DMRs associated with these two genes were hypermethylated in ALS patients compared to controls. The DMR (472 bp, containing 40 CpG sites) associated with ARRB2 is 1–5 kb away from its transcription start site (TSS), suggesting that methylation changes in this region could influence the downstream gene expression. The DMR in CYBA is located within its promoter region and could directly impact the transcriptional activity of its gene. Interestingly, both genes were elevated in ALS patients, but their expression levels decreased in ALS as the disease progressed (Supplementary Fig. 2A-B). This pattern implies their potential involvement in the early stages of ALS pathology. The altered methylation in these regulatory regions of ARRB2 and CYBA could modulate their expression during the disease progression, suggesting that these genes might serve as potential molecular markers for tracking ALS progression.

### Epigenetic footprints in cfDNA reflecting cell-specific gene regulation in ALS

**Fig. 5:**
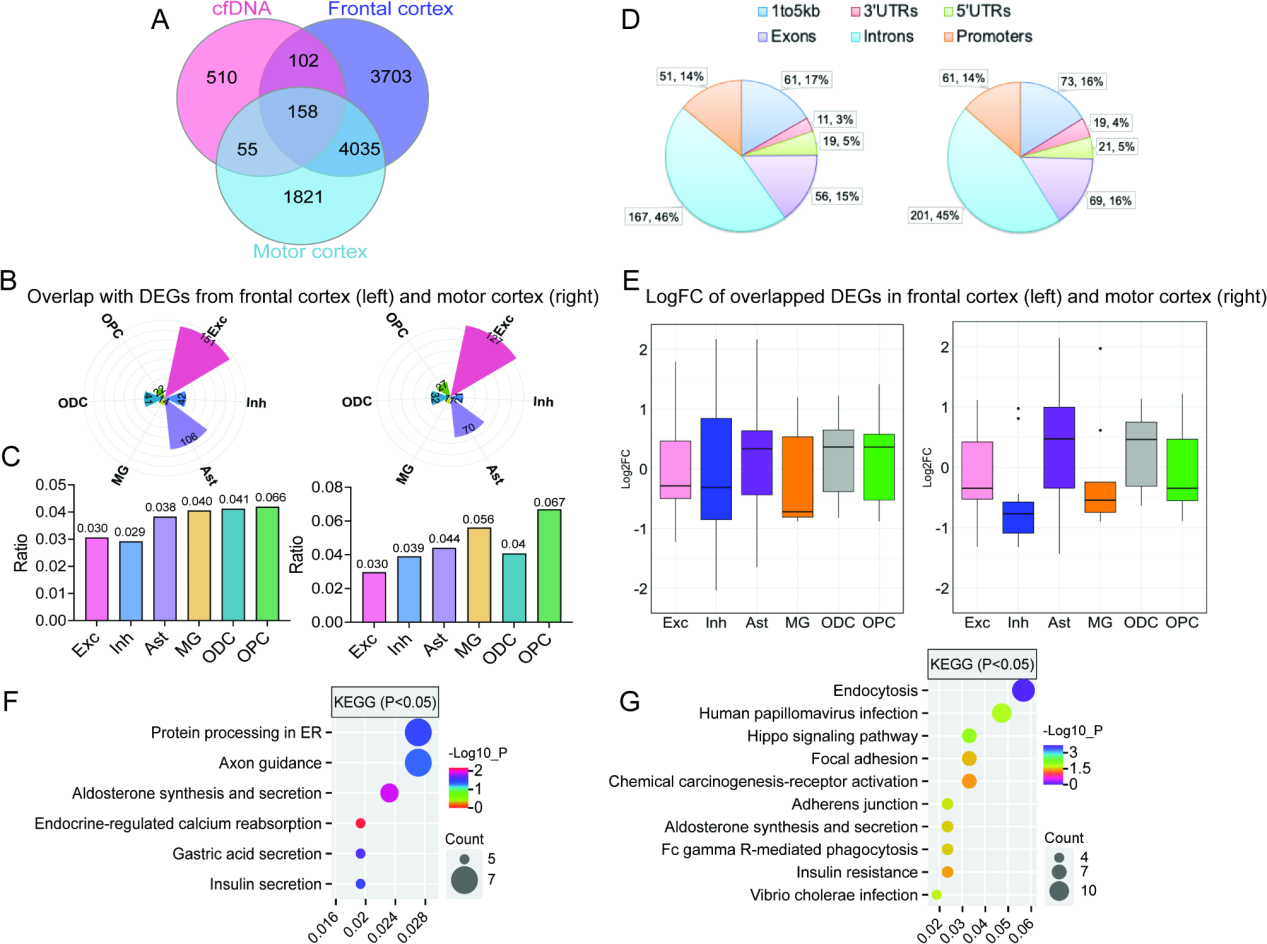
**Integration of cfDNA methylation with snRNA-Seq for frontal cortex and motor cortex of ALS** [32]. **(A)** Venn diagram showing the overlap between cfDNA-derived DMR-associated genes and DEGs identified in ALS patients’ frontal cortex and motor cortex compared to controls. **(B)** Number of DEGs overlapping with DMR-associated genes in six major cell types of the frontal cortex (left) and motor cortex (right), respectively. **(C)** Bar graphs showing the percentage of DEGs in each cell type associated with DMRs in cfDNA. **(D)** Genomic annotation of the DMRs from overlapping genes, showing their percentage of each genomic feature. **(E)** Box plots displaying the LogFC of DEGs with differential methylation in cfDNA across each major cell type in the frontal cortex (left) and motor cortex (right), respectively. **F-G**. KEGG pathway analysis of overlapping genes to assess their functional significance in ALS’s frontal cortex (left) and motor cortex (right)

We next explored whether ALS-related DMRs identified in cfDNA could reflect altered gene regulation at the cellular level in the brain of ALS patients. To address this, we utilized single-nucleus RNA sequencing (snRNA-Seq) data from the frontal and motor cortices of C9orf72 ALS patients [32] to identify genes that exhibited both cfDNA methylation changes and dysregulated gene expression. To ensure consistency with the genome version used in [32], the genomic locations of cfDNA-derived DMRs from ALS patients were converted to hg38, and the annotated genes (*n* = 825) were included in the comparative analysis (Supplementary Table 11). As illustrated in Fig. 5A, we identified 260 DMR-associated genes (31.5%) that were dysregulated across six major cell types in the frontal cortex. In contrast, the motor cortex displayed a smaller overlap, with 203 genes (24.6%) identified (Fig. 5B; Supplementary Tables 12–13). Approximately 19.5% (*n* = 158) of those DMR-associated genes were differentially expressed in both brain regions. Notably, excitatory neurons and astrocytes, both heavily implicated in ALS and displayed significant transcriptional dysregulation in the brains of ALS patients [32], harbor the largest number of dysregulated genes associated with cfDNA methylation changes in both the frontal and motor cortices (Fig. 5B). Other cell types exhibited far fewer overlapping genes between differential methylation and dysregulated expression.

To further characterize these findings, we estimated the percentage of DEGs in each cell type associated with DMRs in cfDNA (Fig. 5C). In the frontal cortex, 3% of DEGs in excitatory neurons exhibited differential methylation, along with 2.9% in inhibitory neurons, 3.8% in astrocytes, 4% in both microglia and oligodendrocytes (ODCs), and 6.6% in oligodendrocyte precursor cells (OPCs). Notably, the motor cortex displayed higher proportions of DEGs with differential methylation in inhibitory neurons (3.9%), astrocytes (4.4%), and microglia (5.6%) compared to the frontal cortex, suggesting that epigenetic regulation in these cell types may differ between brain regions. We also noted that OPCs consistently showed the highest percentage of overlap in both the frontal and motor cortices, highlighting their potential involvement in cfDNA methylation changes related to ALS.

The DMRs detected in these overlapping genes were predominantly located in intronic and promoter regions (Fig. 5D), suggesting that methylation changes in these regulatory regions may contribute to the altered gene expression observed in ALS. Cross-comparison among cell types revealed that most DMR-associated genes were dysregulated in a cell-type-specific manner, especially in excitatory neurons and astrocytes (Supplementary Fig. 3A-B). We then explored the logFC of the identified overlapping DEGs in each cell type. As shown in Fig. 5E (left), in the frontal cortex, the average logFC of DEGs showing cfDNA-derived differential methylation indicated decreased expression in neurons (both excitatory and inhibitory) of ALS patients compared to controls, with similar levels of change between these two cell types. In non-neuronal cells, including astrocytes, ODCs, and OPCs, the average logFC of DEGs demonstrated a trend toward upregulation in ALS, except for the reverse observation in microglia. A similar pattern was observed in the motor cortex (Fig. 5E, right), except in OPCs, where DEGs exhibited reduced expression in ALS-a finding that contrasts with the expression pattern seen in the frontal cortex. We also noted that the gene expression alterations of those DMR-associated genes in excitatory and inhibitory neurons differed in the motor cortex, where the average logFC of DEGs in inhibitory neurons was much lower than that in excitatory neurons. To better capture these differences, we separately examined the logFC of up-regulated and down-regulated DEGs for each cell type (Supplementary Fig. 3C-D). This analysis revealed that inhibitory neurons of motor cortex exhibited a predominant trend toward downregulation, with a magnitude of change compared to upregulated DEGs. Conversely, in non-neuronal cells, particularly astrocytes and ODCs, upregulated DEGs showed greater expression changes than downregulated DEGs. Interestingly, microglia showed regional differences, with most DEGs being downregulated in the motor cortex but showing the opposite trend in the frontal cortex. In addition, we examined the potential correlation between differential methylation levels and LogFC of shared genes in each cell type. However, no significant correlation was observed in any cell type, likely due to limited statistical power (Supplementary Fig. 4). Further KEGG pathway analysis revealed that DEGs showing cfDNA-derived differential methylation in the frontal cortex were significantly enriched in pathways related to protein processing in the ER and axon guidance (Fig. 5F). In contrast, endocytosis was the most enriched pathway for overlapping genes in the motor cortex (Fig. 5G). These observations demonstrated that ALS-associated cfDNA methylation changes could associated with brain region- and cell-type-specific gene regulatory mechanisms underlying ALS pathology.

### Estimation of tissue/cell of origin proportions based on cfDNA methylation

**Fig. 6:**
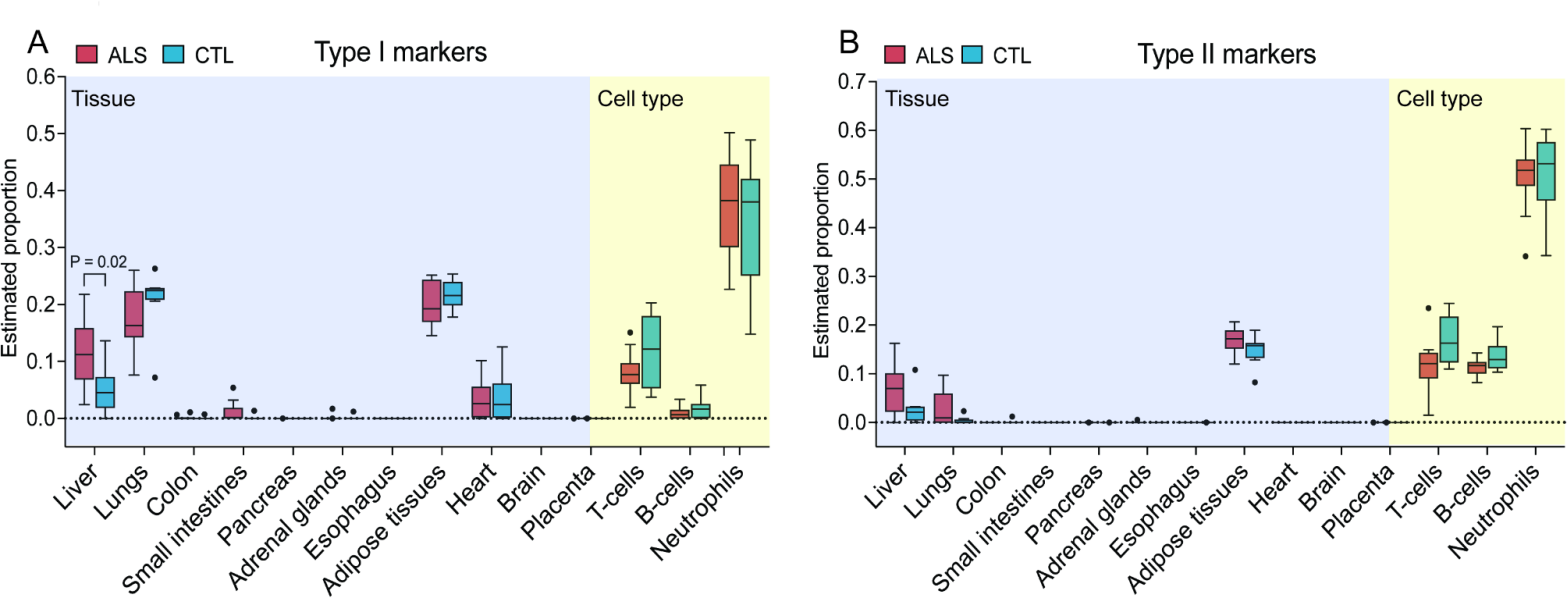
**Box plot for estimated 14 tissue proportions from WGBS data of ALS patients and control subjects using Type I and Type II methylation markers.** ALS patients exhibited a significantly lower proportion of cfDNA from T-cells and B-cells compared to controls, while showed an increased proportion of cfDNA originating from the liver

CfDNA fragments carry epigenetic footprints that reflect their tissue and cellular origins [5]. To estimate these origins, we employed a deconvolution algorithm designed by Sun et al. [41] and optimized by Feng et al. [40]. The reference methylation dataset includes 1,013 type I markers and 4,807 type II methylation markers covering 14 different tissue and cell types [41]. As illustrated in Fig. 6, our analysis revealed that white blood cells, including neutrophils and lymphocytes, are the predominant contributors to the plasma DNA pool. This finding is consistent with established literature indicating that white blood cells are a major source of cfDNA in the bloodstream [40, 56, 57]. Interestingly, we observed a significant reduction in the proportion of cfDNA derived from T-cells and B-cells in ALS patients compared to control individuals. This reduction could indicate altered immune cell dynamics in ALS, potentially due to changes in immune cell populations or their turnover rates in response to the disease. Additionally, our analysis detected cfDNA contributions from non-blood tissues, such as the liver, lungs, heart, and adipose tissue. Notably, ALS patients exhibited an increased proportion of cfDNA originating from the liver, suggesting elevated cell death rates in the liver among ALS patients. Bar plots for estimated tissue proportions for each individual are shown in Supplementary Fig. 5, highlighting a consistent pattern within the ALS cohort.

## Discussion

The methylation signatures of cfDNA from plasma offer a noninvasive diagnostic potential for many diseases and could be a predictive tool for aging. In this study, by leveraging the WGBS of cfDNA, we characterized the epigenetic landscape of cfDNA in the context of aging and ALS. Our findings shed light on the age-dependent methylation signatures in cfDNA and their potential overlap with ALS-related epigenetic changes. Furthermore, we explored the functional implications of ALS-associated DMRs and their relationship with gene expression in ALS-affected tissues. These results support the emerging role of cfDNA as a biomarker for both aging and neurodegenerative diseases like ALS.

Aging is a well-known factor influencing DNA methylation patterns across the genome, which in turn can serve as predictors of biological age and age-related health outcomes [58]. We identified 5,223 DMLs between young and middle-aged adults, with a trend of decreased methylation toward older individuals. These age-related methylation changes may result from various biological mechanisms, including alterations in cellular turnover, organ development, DNA repair processes, and changes in tissue composition over time. Here, we found that many of the identified age-related CpGs in cfDNA were linked to genes involved in cytoskeletal muscle organization and axon guidance. Our findings reinforce the utility of cfDNA in capturing age-related epigenetic alterations. Notably, we observed a significant overlap between cfDNA DML and CpGs identified in large-scale EWAS from blood samples [34], with 3,947 overlapping CpGs (*p* < 0.05). However, only 15 CpGs with FDR < 0.05 were shared across both datasets, likely due to the differing tissue origins of cfDNA and whole-blood DNA. Such discrepancies are expected since cfDNA reflects contributions from various tissues beyond blood cells, including the liver, lungs, heart, and adipose tissue. Despite this, our findings provide candidate loci for future aging research and biomarker discovery.

DNA methylation is increasingly recognized as a significant component of ALS pathology [59], with potential as a biomarker for tracking disease progression and predicting survival outcomes [60]. In this study, we identified 1,045 DMRs between ALS patients and age-matched controls, with slightly more hypomethylated (564) than hypermethylated (481) regions. The enrichment of hypermethylated DMRs in 5’UTR and promoter regions is particularly interesting, as these regions are typically associated with gene repression. This suggests that the hypermethylated DMRs in ALS patients may modulate key genes involved in ALS pathogenesis. Our KEGG pathway analysis further reveals that genes associated with ALS-related DMRs were enriched in pathways related to endocytosis and cell adhesion, which play critical roles in ALS pathology. Impairments in endocytosis and cellular trafficking contribute to the accumulation of toxic protein aggregates, a hallmark of ALS [61–63]. Previous research has also highlighted the importance of dysregulated endocytosis in motor neuron degeneration and neuroinflammation [63–65]. Additionally, dysregulated cell adhesion molecules have been linked to neuronal dysfunction and glial reactivity, further emphasizing the relevance of these pathways in ALS [66, 67]. Our results suggest that cfDNA methylation profiles can offer valuable insights into ALS-specific biological mechanisms.

Aging is a major risk factor for ALS [68]. Previous studies have demonstrated that DNA methylation age acceleration in both blood and CNS tissues correlates with ALS onset and disease duration in C9orf72 mutation carriers and sporadic ALS patients [69, 70]. Our findings build on this by showing that age-related CpGs identified in cfDNA were significantly enriched among ALS-associated DMRs, offering a novel approach to evaluating cfDNA methylation in ALS. This strategy may also aid in predicting the impact of therapeutic interventions on ALS onset and survival.

One of the key findings of our study is the connection between cfDNA methylation changes and gene expression alterations in ALS-affected CNS tissues. By correlating cfDNA-derived DMRs with spinal cord gene expression data, we found significant overlap between DMR-associated genes and DEGs in ALS patients. Moreover, integration with snRNA-seq from ALS brain tissues revealed pronounced overlaps between cfDNA DMR-associated genes and dysregulated genes in excitatory neurons and astrocytes (Fig. 5B). This is consistent with previous studies implicating the critical role of these two cell types in ALS pathogenesis [71, 72]. Dysregulation of excitatory neurons aligns with motor neuron degeneration, a hallmark of ALS, which is a hallmark of ALS [73, 74]. Astrocyte dysregulation reflects their role as mediators of neuroinflammation during ALS progression [75, 76]. Interestingly, the motor cortex exhibited fewer DMR-associated dysregulated genes compared to the frontal cortex but demonstrated higher proportions of DEGs with associated DMRs in specific cell types, including inhibitory neurons, astrocytes, and microglia (Fig. 5C). This may indicate distinct regulatory landscapes across different brain regions affected by ALS, with the motor cortex potentially experiencing more pronounced epigenetic alterations in certain cell populations. Overall, these results highlight that cfDNA captures systemic changes and provides insights into cell-type-specific gene regulation in ALS, further supporting its potential as a non-invasive biomarker for reflecting the complex molecular and cellular alterations in ALS.

Our data demonstrated that cfDNA methylation analysis holds great promise for ALS, particularly in tracking disease progression and therapeutic response. While cfDNA methylation changes have been explored in various diseases, their application in neurodegenerative disorders is still limited. Recent studies have reported cfDNA methylation changes in AD [74, 77, 78], reflecting key neurodegenerative pathways, including synaptic function, neuroinflammation, and autophagy. Our study contributes to this growing body of evidence by demonstrating that ALS-related cfDNA methylation changes are similarly linked to important gene regulatory pathways, particularly endocytosis. This reinforces cfDNA’s potential as a reliable tool for tracking ALS-specific molecular alterations. Specifically, we identified ARRB2 and CYBA as two hypermethylated genes in ALS patients, with their expression levels negatively correlated with disease duration. ARRB2, known to regulate apoptosis and cell survival, plays an essential role in processes disrupted in neurodegenerative diseases like AD, PD and FTD [51–54]. CYBA is involved in the generation of reactive oxygen species, which could play a role in exacerbating neurodegenerative processes [55]. These genes may serve as potential biomarkers or therapeutic targets in ALS, warranting further investigation.

Our deconvolution analysis revealed reduced cfDNA contributions from T-cells and B-cells in ALS patients, likely reflecting alterations in immune cell dynamics, which may relate to the intrinsic innate immunity and inflammatory processes observed in ALS [79]. Immune abnormalities have been detected in ALS patients, with findings in both blood and CSF [80–83]. Additionally, the increased proportion of cfDNA from the liver in ALS patients suggests elevated hepatocyte turnover, which could be indicative of systemic changes associated with ALS pathogenesis. Liver dysfunction, including fatty liver degeneration, has long been associated with ALS [84, 85] and reflect metabolic disturbances observed in motor neuron diseases including ALS [86, 87]. However, our study did not detect significant changes in the proportion of brain-derived cfDNA. This lack of a significant change may indicate that, despite the central role of the brain in ALS pathology, the release of brain-derived cfDNA into the plasma is either not markedly altered or is below the detection threshold of our current methodology. Future studies with larger cohorts and longitudinal designs are required to confirm these findings and to further investigate the potential of cfDNA as a biomarker for ALS.

We acknowledge the limitation of our study that all ALS patients were male. The absence of female ALS patient samples may limit the generalizability of our findings, as sex-specific epigenetic variations could influence the observed methylation changes. However, it is important to note that ALS has a higher prevalence and earlier onset in men, particularly in the younger age groups [88]. Focusing on male patient samples is crucial for understanding the correlation of cfDNA methylation with ALS pathology. Future studies should incorporate both male and female ALS patients to determine whether the identified DMRs and pathway associations are consistent across sexes or if sex-specific differences exist. This would enhance the broader applicability of cfDNA methylation as a biomarker for ALS.

## Conclusions

Overall, our study demonstrated the potential of cfDNA methylation profiles as biomarkers for tracking both age-related epigenetic changes and ALS-associated molecular dysregulation. The significant correlation between cfDNA methylation patterns and gene expression in ALS-affected tissues provides new insights into the utility of cfDNA in capturing tissue- and cell-type-specific gene regulation in ALS and other neurodegenerative diseases. Future research should continue to explore and validate the clinical applicability of cfDNA methylation as a non-invasive tool for monitoring disease progression, early detection, and therapeutic responses in ALS.

## Electronic supplementary material

Below is the link to the electronic supplementary material.

Supplementary Material 1

Supplementary Material 2

## Data Availability

The WGBS datasets of all samples are available from the corresponding authors upon reasonable request.

## References

[1] Peters OM, Ghasemi M, Brown RH. Emerging mechanisms of molecular pathology in ALS. J clin Invest. 2015;125:1767–79. 25932674 10.1172/JCI71601 PMC4463186

[2] Tu S, Vucic S, Kiernan MC. Pathological insights derived from neuroimaging in amyotrophic lateral sclerosis: emerging clinical applications. Curr Opin Neurol. 2024;37:577–84. 38958573 10.1097/WCO.0000000000001295

[3] Lynch K. Pathogenesis and presentation of ALS: examining reasons for delayed diagnosis and identifying opportunities for improvement. Am J Manag Care. 2023;29:S104–11. 37433091 10.37765/ajmc.2023.89390

[4] Jin Y, Allen EG, Jin P. Cell-free DNA methylation as a potential biomarker in brain disorders. Epigenomics. 2022;14:369–74. 35034473 10.2217/epi-2021-0416 PMC9066291

[5] Lo YD, Han DS, Jiang P, Chiu RW. Epigenetics, fragmentomics, and topology of cell-free DNA in liquid biopsies. Science. 2021;372:eaaw3616. 33833097 10.1126/science.aaw3616

[6] Gao Q, Zeng Q, Wang Z, Li C, Xu Y, Cui P et al. Circulating cell-free DNA for cancer early detection. Innov. 2022;3. 10.1016/j.xinn.2022.100259 PMC9133648 35647572

[7] de la Cruz FF, Corcoran R. Methylation in cell-free DNA for early cancer detection. Ann Oncol. 2018;29:1351–3. 29668834 10.1093/annonc/mdy134 PMC6005066

[8] Jamshidi A, Liu MC, Klein EA, Venn O, Hubbell E, Beausang JF, et al. Evaluation of cell-free DNA approaches for multi-cancer early detection. Cancer Cell. 2022;40:1537–49. e1512. 36400018 10.1016/j.ccell.2022.10.022

[9] Chabon JJ, Hamilton EG, Kurtz DM, Esfahani MS, Moding EJ, Stehr H, et al. Integrating genomic features for non-invasive early lung cancer detection. Nature. 2020;580:245–51. 32269342 10.1038/s41586-020-2140-0 PMC8230734

[10] Lo YD, Chan KA, Sun H, Chen EZ, Jiang P, Lun FM, et al. Maternal plasma DNA sequencing reveals the genome-wide genetic and mutational profile of the fetus. Sci Trans Med. 2010;2:61ra91. 10.1126/scitranslmed.3001720 21148127

[11] Norton ME, Baer RJ, Wapner RJ, Kuppermann M, Jelliffe-Pawlowski LL, Currier RJ. Cell-free DNA vs sequential screening for the detection of fetal chromosomal abnormalities. Am J Obstet Gynecol. 2016;214(727):e721–6. 10.1016/j.ajog.2015.12.018 26709085

[12] Zhao C, Tynan J, Ehrich M, Hannum G, McCullough R, Saldivar J-S, et al. Detection of fetal subchromosomal abnormalities by sequencing circulating cell-free DNA from maternal plasma. Clin Chem. 2015;61:608–16. 25710461 10.1373/clinchem.2014.233312

[13] Zhang J, Wu Y, Chen S, Luo Q, Xi H, Li J, et al. Prospective prenatal cell-free DNA screening for genetic conditions of heterogenous etiologies. Nat Med. 2024;30:470–9. 38253798 10.1038/s41591-023-02774-x

[14] Shea JL, Diamandis EP, Hoffman B, Lo YD, Canick J, Van Den Boom D. A new era in prenatal diagnosis: the use of cell-free fetal DNA in maternal circulation for detection of chromosomal aneuploidies. Clin Chem. 2013;59:1151–9. 23426426 10.1373/clinchem.2012.201996

[15] Drury S, Hill M, Chitty L. Cell-free fetal DNA testing for prenatal diagnosis. Adv Clin Chem. 2016;76:1–35. 27645814 10.1016/bs.acc.2016.05.004

[16] Vora NL, Langlois S, Chitty LS. Current controversy in prenatal diagnosis: the use of cfDNA to screen for monogenic conditions in low risk populations is ready for clinical use. Prenat Diagn. 2024;44:389–97. 37991340 10.1002/pd.6469

[17] Hu Y, Zhao Y, Zhang Y, Chen W, Zhang H, Jin X. Cell-free DNA: a promising biomarker in infectious diseases. Trends Microbiol. 2024. 10.1016/j.tim.2024.06.005 38997867

[18] Fernández-Carballo BL, Broger T, Wyss R, Banaei N, Denkinger CM. Toward the development of a circulating free DNA-based in vitro diagnostic test for infectious diseases: a review of evidence for tuberculosis. J Clin Microbiol. 2019;57:e01234–01218. 30404942 10.1128/JCM.01234-18 PMC6440766

[19] Blauwkamp TA, Thair S, Rosen MJ, Blair L, Lindner MS, Vilfan ID, et al. Analytical and clinical validation of a microbial cell-free DNA sequencing test for infectious disease. Nat Microbiol. 2019;4:663–74. 30742071 10.1038/s41564-018-0349-6

[20] Knight SR, Thorne A, Faro MLL. Donor-specific cell-free DNA as a biomarker in solid organ transplantation. A systematic review. Transplantation. 2019;103:273–83. 30308576 10.1097/TP.0000000000002482

[21] Lui YY, Woo K-S, Wang AY, Yeung C-K, Li PK, Chau E, et al. Origin of plasma cell-free DNA after solid organ transplantation. Clin Chem. 2003;49:495–6. 12600963 10.1373/49.3.495

[22] Beck J, Oellerich M, Schulz U, Schauerte V, Reinhard L, Fuchs U et al. Donor-derived cell-free DNA is a novel universal biomarker for allograft rejection in solid organ transplantation. Transplant Proc. 2015: 2400–2403. 10.1016/j.transproceed.2015.08.035 26518940

[23] Luo H, Wei W, Ye Z, Zheng J, Xu R-h. Liquid biopsy of methylation biomarkers in cell-free DNA. Trends Mol Med. 2021;27:482–500. 33500194 10.1016/j.molmed.2020.12.011

[24] Dolinar A, Ravnik-Glavač M, Glavač D. Epigenetic mechanisms in amyotrophic lateral sclerosis: a short review. Mech Ageing Dev. 2018;174:103–10. 29545202 10.1016/j.mad.2018.03.005

[25] Hop PJ, Zwamborn RA, Hannon E, Shireby GL, Nabais MF, Walker EM, et al. Genome-wide study of DNA methylation shows alterations in metabolic, inflammatory, and cholesterol pathways in ALS. Sci Transl Med. 2022;14:eabj0264. 35196023 10.1126/scitranslmed.abj0264 PMC10040186

[26] Garton FC, Benyamin B, Zhao Q, Liu Z, Gratten J, Henders AK, et al. Whole exome sequencing and DNA methylation analysis in a clinical amyotrophic lateral sclerosis cohort. Mol Genet Genomic. 2017;5:418–28. 10.1002/mgg3.302 PMC5511806 28717666

[27] Coppedè F, Stoccoro A, Mosca L, Gallo R, Tarlarini C, Lunetta C, et al. Increase in DNA methylation in patients with amyotrophic lateral sclerosis carriers of not fully penetrant SOD1 mutations. Amyotroph Lateral Scler Frontotemporal Degener. 2018;19:93–101. 28859526 10.1080/21678421.2017.1367401

[28] Martin LJ, Wong M. Aberrant regulation of DNA methylation in amyotrophic lateral sclerosis: a new target of disease mechanisms. Neurotherapeutics. 2013;10:722–33. 23900692 10.1007/s13311-013-0205-6 PMC3805862

[29] Appleby-Mallinder C, Schaber E, Kirby J, Shaw PJ, Cooper‐Knock J, Heath PR, et al. TDP43 proteinopathy is associated with aberrant DNA methylation in human amyotrophic lateral sclerosis. Neuropathol Appl Neurobiol. 2021;47:61–72. 32365404 10.1111/nan.12625

[30] Tremolizzo L, Messina P, Conti E, Sala G, Cecchi M, Airoldi L, et al. Whole-blood global DNA methylation is increased in amyotrophic lateral sclerosis independently of age of onset. Amyotroph Lateral Scler Frontotemporal Degener. 2014;15:98–105. 24224837 10.3109/21678421.2013.851247

[31] Morahan JM, Yu B, Trent RJ, Pamphlett R. A genome-wide analysis of brain DNA methylation identifies new candidate genes for sporadic amyotrophic lateral sclerosis. Amyotroph Lateral Scler. 2009;10:418–29. 19922134 10.3109/17482960802635397

[32] Li J, Jaiswal MK, Chien J-F, Kozlenkov A, Jung J, Zhou P, et al. Divergent single cell transcriptome and epigenome alterations in ALS and FTD patients with C9orf72 mutation. Nat Comm. 2023;14:5714. 10.1038/s41467-023-41033-y PMC10504300 37714849

[33] Pineda SS, Lee H, Ulloa-Navas MJ, Linville RM, Garcia FJ, Galani K, et al. Single-cell dissection of the human motor and prefrontal cortices in ALS and FTLD. Cell. 2024;187:1971–89. e1916. 38521060 10.1016/j.cell.2024.02.031 PMC11086986

[34] Bernabeu E, McCartney DL, Gadd DA, Hillary RF, Lu AT, Murphy L, et al. Refining epigenetic prediction of chronological and biological age. Genome Med. 2023;15:12. 36855161 10.1186/s13073-023-01161-y PMC9976489

[35] Bolger AM, Lohse M, Usadel B. Trimmomatic: a flexible trimmer for Illumina sequence data. Bioinformatics. 2014;30:2114–20. 24695404 10.1093/bioinformatics/btu170 PMC4103590

[36] Krueger F, Andrews SR. Bismark: a flexible aligner and methylation caller for Bisulfite-Seq applications. Bioinformatics. 2011;27:1571–2. 21493656 10.1093/bioinformatics/btr167 PMC3102221

[37] Feng H, Wu H. Differential methylation analysis for bisulfite sequencing using DSS. Quant Biol. 2019;7:327–34. 34084562 10.1007/s40484-019-0183-8 PMC8171293

[38] Cavalcante RG, Sartor MA. Annotatr: genomic regions in context. Bioinformatics. 2017;33:2381–3. 28369316 10.1093/bioinformatics/btx183 PMC5860117

[39] Sherman BT, Hao M, Qiu J, Jiao X, Baseler MW, Lane HC, et al. DAVID: a web server for functional enrichment analysis and functional annotation of gene lists (2021 update). Nucleic Acids Res. 2022;50:W216–21. 35325185 10.1093/nar/gkac194 PMC9252805

[40] Feng H, Jin P, Wu H. Disease prediction by cell-free DNA methylation. Brief Bioinform. 2019;20:585–97. 29672679 10.1093/bib/bby029 PMC6556903

[41] Sun K, Jiang P, Chan KA, Wong J, Cheng YK, Liang RH, et al. Plasma DNA tissue mapping by genome-wide methylation sequencing for noninvasive prenatal, cancer, and transplantation assessments. Proc Natl Acad Sci USA. 2015;112:E5503–12. 26392541 10.1073/pnas.1508736112 PMC4603482

[42] Ramírez F, Ryan DP, Grüning B, Bhardwaj V, Kilpert F, Richter AS, et al. deepTools2: a next generation web server for deep-sequencing data analysis. Nucleic Acids Res. 2016;44:W160. 27079975 10.1093/nar/gkw257 PMC4987876

[43] Zerbino DR, Johnson N, Juettemann T, Wilder SP, Flicek P. WiggleTools: parallel processing of large collections of genome-wide datasets for visualization and statistical analysis. Bioinformatics. 2014;30:1008–9. 24363377 10.1093/bioinformatics/btt737 PMC3967112

[44] Wheater EN, Stoye DQ, Cox SR, Wardlaw JM, Drake AJ, Bastin ME, et al. DNA methylation and brain structure and function across the life course: a systematic review. Neurosci Biobehav Rev. 2020;113:133–56. 32151655 10.1016/j.neubiorev.2020.03.007 PMC7237884

[45] Mo A, Mukamel EA, Davis FP, Luo C, Henry GL, Picard S, et al. Epigenomic signatures of neuronal diversity in the mammalian brain. Neuron. 2015;86:1369–84. 26087164 10.1016/j.neuron.2015.05.018 PMC4499463

[46] Loyfer N, Magenheim J, Peretz A, Cann G, Bredno J, Klochendler A, et al. A DNA methylation atlas of normal human cell types. Nature. 2023;613:355–64. 36599988 10.1038/s41586-022-05580-6 PMC9811898

[47] Jin Y, Su K, Kong HE, Ma W, Wang Z, Li Y, et al. Cell type-specific DNA methylome signatures reveal epigenetic mechanisms for neuronal diversity and neurodevelopmental disorder. Hum Mol Genet. 2023;32:218–30. 35947991 10.1093/hmg/ddac189 PMC9840206

[48] Sun K, Jiang P, Cheng SH, Cheng THT, Wong J, Wong VWS, et al. Orientation-aware plasma cell-free DNA fragmentation analysis in open chromatin regions informs tissue of origin. Genome Res. 2019;29:418–27. 30808726 10.1101/gr.242719.118 PMC6396422

[49] Humphrey J, Venkatesh S, Hasan R, Herb JT, de Paiva Lopes K, Küçükali F, et al. Integrative transcriptomic analysis of the amyotrophic lateral sclerosis spinal cord implicates glial activation and suggests new risk genes. Nat Neurosci. 2023;26:150–62. 36482247 10.1038/s41593-022-01205-3

[50] Hardiman O, Al-Chalabi A, Chio A, Corr EM, Logroscino G, Robberecht W, et al. Amyotrophic lateral sclerosis. Nat Rev Dis Primers. 2017;3:17071. 28980624 10.1038/nrdp.2017.71

[51] Thathiah A, Horré K, Snellinx A, Vandewyer E, Huang Y, Ciesielska M, et al. β-arrestin 2 regulates Aβ generation and γ-secretase activity in Alzheimer’s disease. Nat Med. 2013;19:43–9. 23202293 10.1038/nm.3023

[52] Jiang T, Yu JT, Tan MS, Zhu XC, Tan L. β-Arrestins as potential therapeutic targets for Alzheimer’s disease. Mol Neurobiol. 2013;48:812–8. 23677646 10.1007/s12035-013-8469-8

[53] Thathiah A. β-Arrestin2 arrests the clearance of tau in FTLD. Proc Natl Acad Sci USA. 2020;117:6968–70. 32188777 10.1073/pnas.2001455117 PMC7132289

[54] Fang Y, Jiang Q, Li S, Zhu H, Xu R, Song N, et al. Opposing functions of β-arrestin 1 and 2 in Parkinson’s disease via microglia inflammation and Nprl3. Cell Death Differ. 2021;28:1822–36. 33686256 10.1038/s41418-020-00704-9 PMC8184754

[55] Barber SC, Mead RJ, Shaw PJ. Oxidative stress in ALS: a mechanism of neurodegeneration and a therapeutic target. Biochim Biophys Acta. 2006;1762:1051–67. 16713195 10.1016/j.bbadis.2006.03.008

[56] Caggiano C, Celona B, Garton F, Mefford J, Black BL, Henderson R, et al. Comprehensive cell type decomposition of circulating cell-free DNA with CelFiE. Nat Commun. 2021;12:2717. 33976150 10.1038/s41467-021-22901-x PMC8113516

[57] Moss J, Magenheim J, Neiman D, Zemmour H, Loyfer N, Korach A, et al. Comprehensive human cell-type methylation atlas reveals origins of circulating cell-free DNA in health and disease. Nat Commun. 2018;9:5068. 30498206 10.1038/s41467-018-07466-6 PMC6265251

[58] Noroozi R, Ghafouri-Fard S, Pisarek A, Rudnicka J, Spólnicka M, Branicki W, et al. DNA methylation-based age clocks: from age prediction to age reversion. Ageing Res Rev. 2021;68:101314. 33684551 10.1016/j.arr.2021.101314

[59] Jimenez-Pacheco A, Franco JM, Lopez S, Gomez-Zumaquero JM, Magdalena Leal-Lasarte M, Caballero-Hernandez DE et al. Epigenetic mechanisms of gene regulation in amyotrophic lateral sclerosis. Adv Exp Med Biol. 2017;255–75. 10.1007/978-3-319-53889-1_14 28523551

[60] Yang T, Li C, Wei Q, Pang D, Cheng Y, Huang J, et al. Genome-wide DNA methylation analysis related to ALS patient progression and survival. J Neurol. 2024;271:2672–83. 38372747 10.1007/s00415-024-12222-6

[61] Burk K, Pasterkamp RJ. Disrupted neuronal trafficking in amyotrophic lateral sclerosis. Act Neuropathol. 2019;137:859–77. 10.1007/s00401-019-01964-7 PMC6531423 30721407

[62] Liu G, Coyne AN, Pei F, Vaughan S, Chaung M, Zarnescu DC, et al. Endocytosis regulates TDP-43 toxicity and turnover. Nat Commun. 2017;8:2092. 29233983 10.1038/s41467-017-02017-x PMC5727062

[63] Schreij AM, Fon EA, McPherson PS. Endocytic membrane trafficking and neurodegenerative disease. Cell Mol Life Sci. 2016;73:1529–45. 26721251 10.1007/s00018-015-2105-x PMC11108351

[64] Cosker KE, Segal RA. Neuronal signaling through endocytosis. Cold Spring Harbor Perspect biol. 2014;6:a020669. 10.1101/cshperspect.a020669 PMC3941234 24492712

[65] Kuijpers M, Haucke V. Presynaptic endocytic factors in autophagy and neurodegeneration. Curr Opin Neurol. 2018;48:153–9. 10.1016/j.conb.2017.12.018 29316491

[66] Wielgat P, Braszko J. Significance of the cell adhesion molecules and sialic acid in neurodegeneration. Adv Med Sci. 2012;57:23–30. 22440941 10.2478/v10039-012-0011-0

[67] Chen Q, Peto CA, Shelton GD, Mizisin A, Sawchenko PE, Schubert D. Loss of modifier of cell adhesion reveals a pathway leading to axonal degeneration. J Neurosci. 2009;29:118–30. 19129390 10.1523/JNEUROSCI.3985-08.2009 PMC2669744

[68] Logroscino G, Tortelli R, Rizzo G, Marin B, Preux PM, Malaspina A. Amyotrophic lateral sclerosis: an aging-related disease. Curr Geriatr Rep. 2015;4:142–53.

[69] Zhang M, McKeever PM, Xi Z, Moreno D, Sato C, Bergsma T, et al. DNA methylation age acceleration is associated with ALS age of onset and survival. Acta Neuropathol. 2020;139:943–6. 32146547 10.1007/s00401-020-02131-z PMC7181538

[70] Zhang M, Tartaglia MC, Moreno D, Sato C, McKeever P, Weichert A, et al. DNA methylation age-acceleration is associated with disease duration and age at onset in C9orf72 patients. Acta Neuropathol. 2017;134:271–9. 28439722 10.1007/s00401-017-1713-y PMC5508035

[71] Van Den Bosch L, Van Damme P, Bogaert E, Robberecht W. The role of excitotoxicity in the pathogenesis of amyotrophic lateral sclerosis. Biochim Biophys Acta Mol Basis Dis. 2006;1762:1068–82. 10.1016/j.bbadis.2006.05.002 16806844

[72] Yamanaka K, Komine O. The multi-dimensional roles of astrocytes in ALS. Neurosci Res. 2018;126:31–8. 29054467 10.1016/j.neures.2017.09.011

[73] Gunes ZI, Kan VW, Ye X, Liebscher S. Exciting complexity: the role of motor circuit elements in ALS pathophysiology. Front Neurosci. 2020;14:573. 32625051 10.3389/fnins.2020.00573 PMC7311855

[74] King AE, Woodhouse A, Kirkcaldie MT, Vickers JC. Excitotoxicity in ALS: overstimulation, or overreaction? Exp Neurol. 2016;275:162–71. 26584004 10.1016/j.expneurol.2015.09.019

[75] Vaz SH, Pinto S, Sebastião AM, Brites D. Astrocytes in amyotrophic lateral sclerosis. Exon Publications. 2021;35–53. 34473439

[76] Provenzano F, Torazza C, Bonifacino T, Bonanno G, Milanese M. The key role of astrocytes in amyotrophic lateral sclerosis and their commitment to glutamate excitotoxicity. Int J Mol Sci. 2023;24:15430. 37895110 10.3390/ijms242015430 PMC10607805

[77] Bahado-Singh RO, Radhakrishna U, Gordevičius J, Aydas B, Yilmaz A, Jafar F, et al. Artificial intelligence and circulating cell-free DNA methylation profiling: mechanism and detection of Alzheimer’s disease. Cells. 2022;11:1744. 35681440 10.3390/cells11111744 PMC9179874

[78] Ding B, Zhang X, Wan Z, Tian F, Ling J, Tan J, et al. Characterization of mitochondrial DNA methylation of alzheimer’s disease in plasma cell-free DNA. Diagnostics. 2023;13:2351. 37510095 10.3390/diagnostics13142351 PMC10378411

[79] A McCombe P, Henderson D. The role of immune and inflammatory mechanisms in ALS. Curr Mol Med. 2011;11:246–54. 21375489 10.2174/156652411795243450 PMC3182412

[80] Hovden H, Frederiksen J, Pedersen S. Immune system alterations in amyotrophic lateral sclerosis. Acta Neurol Scand. 2013;128:287–96. 23550891 10.1111/ane.12125

[81] Murdock BJ, Zhou T, Kashlan SR, Little RJ, Goutman SA, Feldman EL. Correlation of peripheral immunity with rapid amyotrophic lateral sclerosis progression. JAMA Neurol. 2017;74:1446–54. 28973548 10.1001/jamaneurol.2017.2255 PMC5822195

[82] Grassano M, Manera U, De Marchi F, Cugnasco P, Matteoni E, Daviddi M, et al. The role of peripheral immunity in ALS: a population-based study. Ann Clin Transl Neurol. 2023;10:1623–32. 37482930 10.1002/acn3.51853 PMC10502618

[83] Puentes F, Malaspina A, Van Noort JM, Amor S. Non-neuronal cells in ALS: role of glial, immune cells and blood‐CNS barriers. Brain Pathol. 2016;26:248–57. 26780491 10.1111/bpa.12352 PMC8029111

[84] Nakano Y, Hirayama K, Terao K. Hepatic ultrastructural changes and liver dysfunction in amyotrophic lateral sclerosis. Arch Neurol. 1987;44:103–6. 3800708 10.1001/archneur.1987.00520130079022

[85] Fisman M. Hepatic ultrastructural change and liver dysfunction in amyotrophic lateral sclerosis. Arch Neurol. 1987;44:997–997. 3632388 10.1001/archneur.1987.00520220007005

[86] Nodera H, Takamatsu N, Muguruma N, Ukimoto K, Nishio S, Oda M, et al. Frequent hepatic steatosis in amyotrophic lateral sclerosis: implication for systemic involvement. Neurol Clin Neurosci. 2015;3:58–62.

[87] Zolkipli Z, Sherlock M, Biggar WD, Taylor G, Hutchison JS, Peliowski A, et al. Abnormal fatty acid metabolism in spinal muscular atrophy may predispose to perioperative risks. Eur J Paediatr Neurol. 2012;16:549–53. 22264649 10.1016/j.ejpn.2012.01.004

[88] Manjaly ZR, Scott KM, Abhinav K, Wijesekera L, Ganesalingam J, Goldstein LH et al. 2010. The sex ratio in amyotrophic lateral sclerosis: A population based study. Amyotroph Lateral Scler. 2010;11:439–442. 10.3109/17482961003610853 PMC6485484 20225930

