# Supplementary material for: Whole-genome bisulfite sequencing of cell-free DNA unveils age-dependent and ALS-associated methylation alterations
Source: Cell Biosci. 2025 Feb 20;15:26. doi: 10.1186/s13578-025-01366-1 (PMC11843967; doi:10.1186/s13578-025-01366-1)
Supplement: Supplementary file 2 — Supplementary Material 2 [file 13578_2025_1366_MOESM2_ESM.pdf]

Figure S1

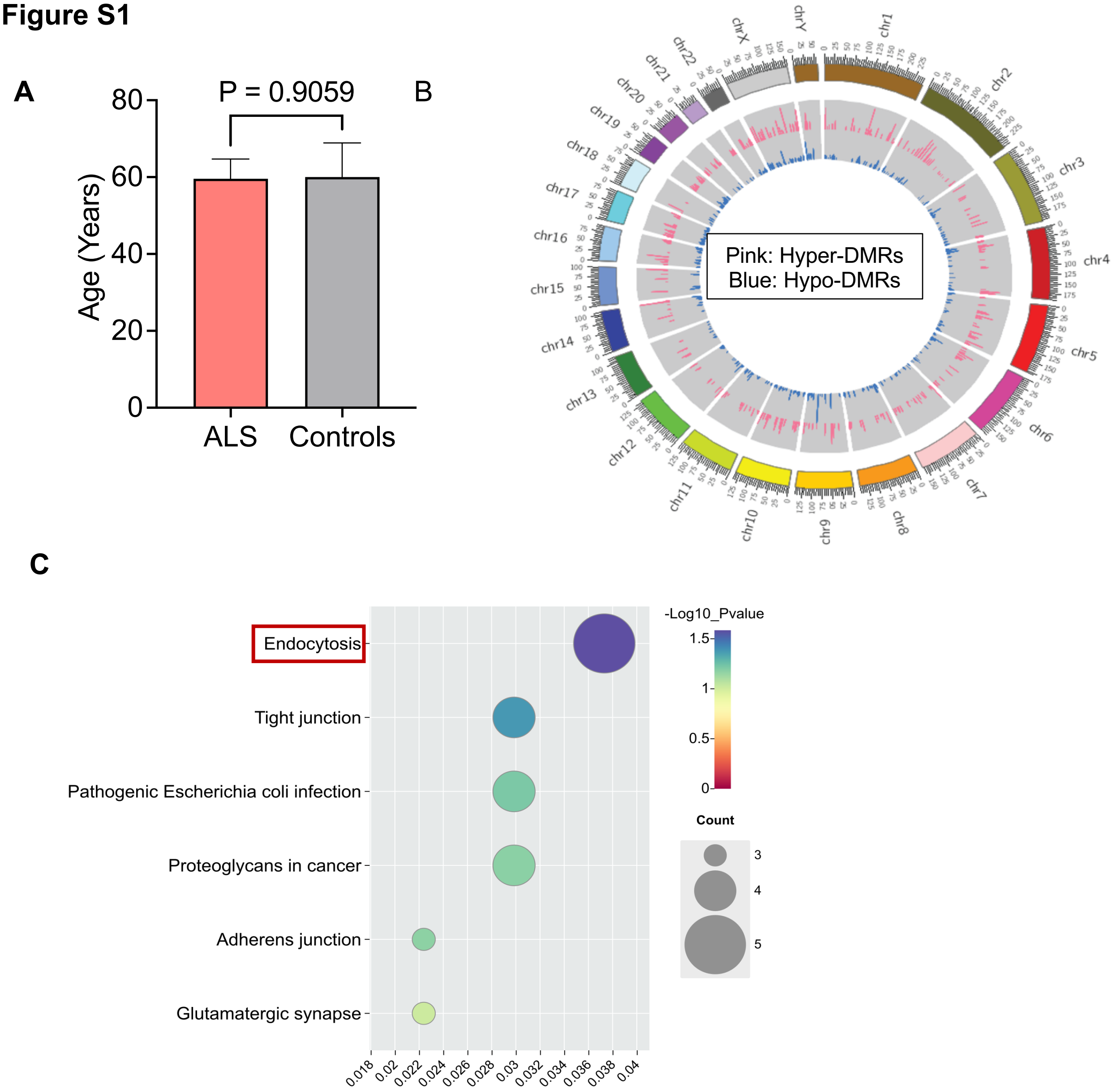

A. Bar graph displaying the age distribution of ALS patients and control subjects. **B**. Distribution of ALS-related DMRs across each chromosome in the human genome. **C**. KEGG pathway analysis revealing significant enrichment of the endocytosis pathway in genes associated with promoter-region DMRs.

Figure S2

A

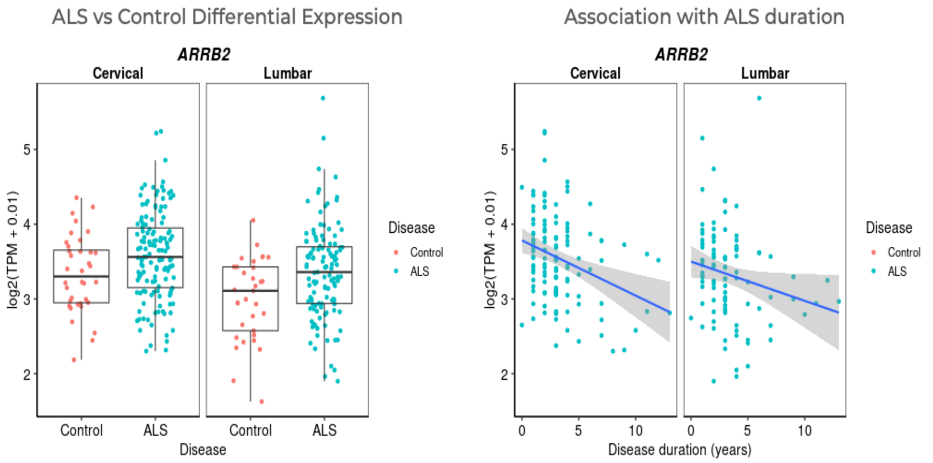

	Region	log2 fold change	P value	FDR
1	Cervical	0.62	9.6e-11	1.1e-8
2	Lumbar	0.55	3.6e-7	0.000026

	Region	log2 fold change	P value	FDR
1	Cervical	-0.0052	0.0000099	0.0027
2	Lumbar	-0.0057	0.000076	0.05

B

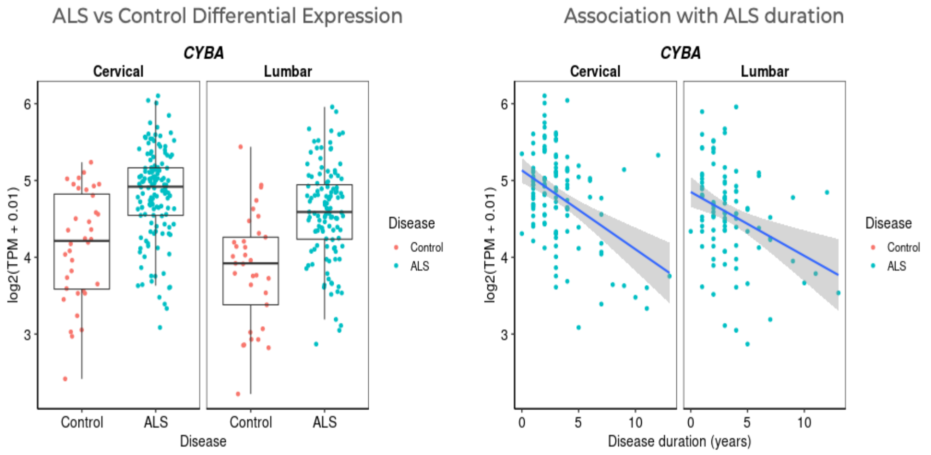

	Region	log2 fold change	P value	FDR
1	Cervical	0.87	2e-11	3.3e-9
2	Lumbar	0.74	6e-7	0.000037

	Region	log2 fold change	P value	FDR
1	Cervical	-0.0068	0.0000047	0.002
2	Lumbar	-0.0074	0.000045	0.041

A-B. Differential expression profiles of ARRB2 (A) and CYBA (B) in the cervical and lumbar spinal segments of ALS patients, with an analysis of their correlation to ALS disease duration. The figures were directly sourced from the ALS Spinal Cord Browser (https://rstudio-connect.hpc.mssm.edu/als_spinal_cord_browser/) developed by Humphrey et al. (2023).

Figure S3

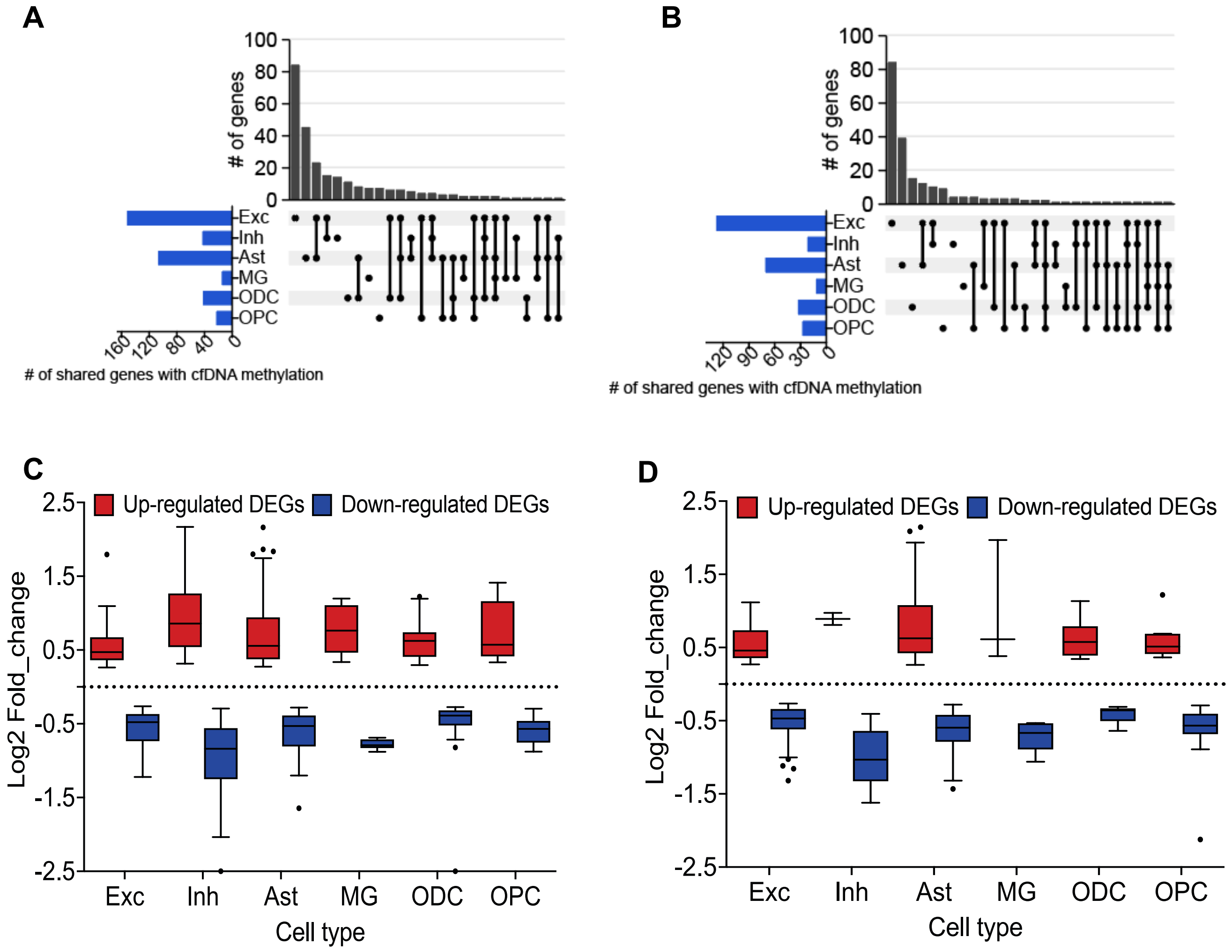

A-B. Upset plots comparing DMR-related genes with differential expression across six major cell types in the frontal cortex (A) and motor cortex (B). Box plots displaying the Log2 fold change of DEGs overlapped with DMR-associated genes by separating the visualization of up-regulated and down-regulated DEGs in the frontal cortex (C) and motor cortex (D).

Figure S4

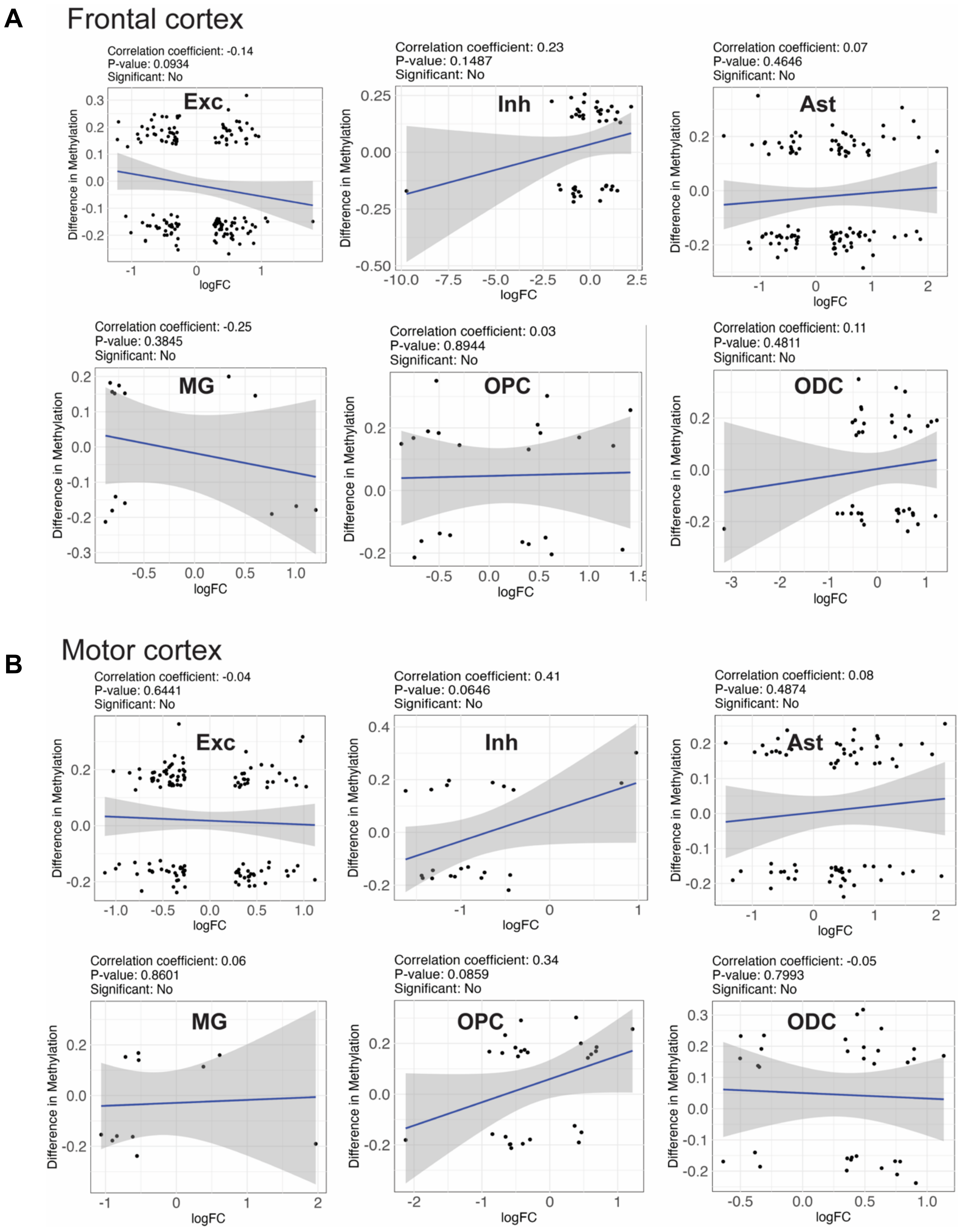

A-B. Correlation analysis between differential methylation levels and LogFC of shared genes in major cell types of the frontal cortex (A) and motor cortex (B) in ALS patients. No significant correlation was observed in any cell type.

Figure S5

A. Type I markers per sample

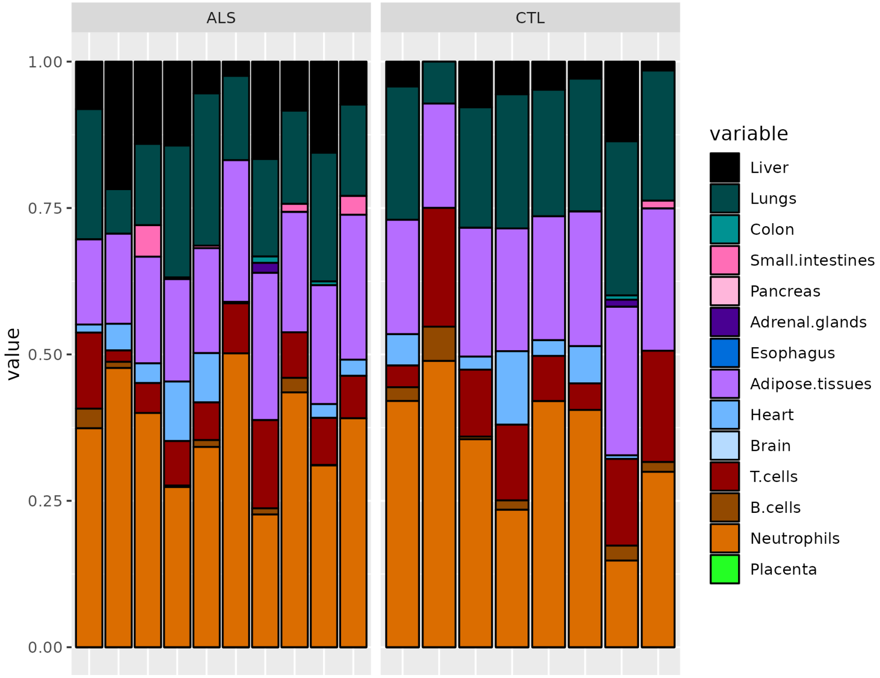

B. Type II markers per sample

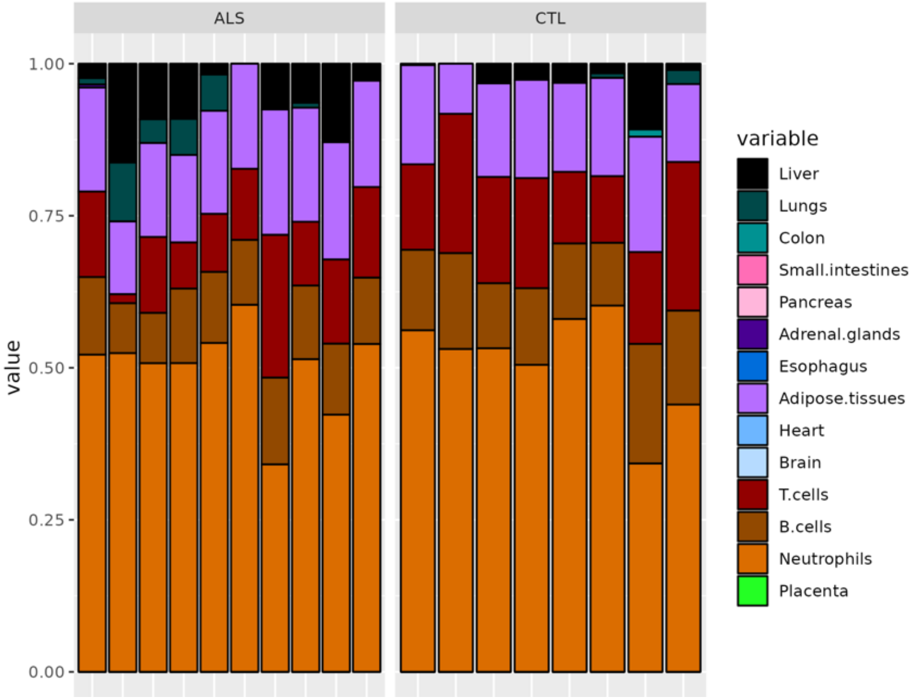

A-B. Bar plots for estimated tissue proportions in each individual by using Type I (A) and Type II (B) methylation markers.
